# Tissue factor promotes vascular invasion and metastasis in hepatocellular carcinoma via combined activation of β-catenin/STAT3 signaling

**DOI:** 10.1186/s43556-026-00471-y

**Published:** 2026-05-23

**Authors:** Wen-chao Wang, Wei-dan Ji, Jun-yong Ma, Yun Pan, Lei Chen, Xue-jing Lin, Ying Chen, Min Tang, Hai-long Liu, Mou-bin Lin, Xiao-feng Zhang, Bin Sun

**Affiliations:** 1 Department of General Surgery, Yangpu Hospital, Tongji University School of Medicine, Shanghai, 200090 People’s Republic of China; 2 National Center for Liver Cancer, Navy Military Medical University, Shanghai, 200438 People’s Republic of China; 3 Department of Hepatic Surgery, Eastern Hepatobiliary Surgery Hospital, Navy Military Medical University, Shanghai, 200438 People’s Republic of China; 4 Center for Clinical Research and Translational Medicine, Yangpu Hospital, Tongji University School of Medicine, Shanghai, 200090 People’s Republic of China

**Keywords:** Hepatocellular carcinoma, Tissue factor, Circulating tumor cell/microemboli, Microvascular invasion, Portal vein tumor thrombus, Metastasis

## Abstract

Vascular invasion critically determines intrahepatic and extrahepatic metastasis and early recurrence in hepatocellular carcinoma (HCC), yet its underlying mechanisms remain poorly defined. In this study, we employed EdU proliferation, colony formation, wound healing, transwell, spheroid formation, and immunofluorescence assays, along with xenograft tumor and liver/lung metastasis models, immunohistochemistry, and western blotting to investigate the biological functions of tissue factor (TF) and the therapeutic potential of targeting TF and its downstream signaling. Our results demonstrate that TF is upregulated in HCC tissues and correlates with poor prognosis. TF overexpression significantly enhanced HCC cell proliferation, migration, invasion, and spheroid formation in vitro, and promoted tumor growth and metastasis in vivo. Clinical sample analysis further revealed that high TF expression increases the dissemination potential of circulating tumor cells (CTCs) and circulating tumor microemboli (CTMs) in peripheral blood, thereby facilitating hematogenous spread. Mechanistically, TF activates PAR1 on HCC cell surfaces, leading to activation of both the Wnt/β-catenin and JAK2/STAT3 pathways, with nuclear translocation and accumulation of β-catenin and STAT3. Notably, nuclear STAT3 enhances TF promoter activity, forming a positive feedback loop that sustains downstream signaling. TF also promotes extracellular matrix degradation. Importantly, combined inhibition of TF with β-catenin and STAT3 blockers effectively suppressed HCC tumorigenesis and metastasis both in vitro and in vivo. These findings elucidate the oncogenic role of TF in vascular invasion-mediated HCC metastasis and suggest a promising combinatorial therapeutic strategy for HCC treatment.

**Supplementary Information:**

The online version contains supplementary material available at 10.1186/s43556-026-00471-y.

## Introduction

Hepatocellular carcinoma (HCC) is a highly vascular malignant tumor. Tumor thrombus formation and vascular invasion are common malignant biological events that frequently leads to intrahepatic metastasis and early recurrence. These processes represent major obstacles to effective HCC treatment and are closely associated with poor patient prognosis [1, 2]. Vascular invasion in HCC is generally classified into macrovascular invasion or microvascular invasion (MVI).

MVI is defined as the presence of cancer cell nests within the lumen of small, endothelial-lined blood vessels, and can only be detected microscopically [3]. MVI most commonly occurs in small branches of the portal vein within the tumor capsule and adjacent liver tissue. As an intermediate stage in HCC progression, MVI has the potential to evolve into large-vessel invasion or portal vein tumor thrombus (PVTT), thereby facilitating intrahepatic dissemination and distant metastasis [2, 4, 5].

To date, the causes and mechanisms underlying vascular invasion in HCC remain unclear. Previous studies have suggested that MVI results from the combined effects of multiple factors, including alterations in pathological anatomy, hemodynamics, and molecular biology associated with liver cirrhosis [6, 7]. More recent evidence indicates that vascular invasion may arise when circulating tumor cells (CTCs) enter the bloodstream, adhere to the vascular endothelium, and subsequently proliferate to form cancer cell nests or tumor thrombi within microvessels or the portal venous system [4, 6, 8]. However, few studies have clearly elucidated the dominant role of HCC-derived CTCs or the molecular mechanisms by which they contribute to the development of MVI or PVTT.

Tissue factor (TF) is the only type I transmembrane protein among coagulation factors expressed on the cell membrane. As a cell surface receptor for coagulation factor VII (FVII), TF binds and activates FVII to form the TF/FVIIa complex, which triggers the extrinsic coagulation cascade through activation of protease-activated receptors (PARs) on various cell types [9, 10]. TF has been shown to be highly expressed in multiple tumor types and is associated with poor prognosis in HCC, breast cancer, colorectal cancer, and pancreatic cancer [11–16]. Based on these findings, we hypothesize that CTCs released from primary HCC lesions may also exhibit high TF expression. This may lead to FVII activation, initiation of the extrinsic coagulation pathway, promotion of CTC aggregation, and ultimately the formation of CTC clusters or circulating tumor microemboli (CTM).

In this study, we investigated the processes and mechanisms by which TF promotes thrombus formation in HCC using a series of in vitro and in vivo experiments. We found that HCC patients with high TF expression exhibit an increased propensity to release CTCs. After entering the bloodstream, these CTCs activate the extrinsic coagulation pathway, resulting in CTM formation. Mechanistically, TF predominantly activates PAR1, which in turn stimulates the Wnt/β-catenin and JAK2/STAT3 signaling pathways, thereby enhancing HCC cell proliferation and adhesion. This cascade ultimately facilitates vascular invasion. Collectively, these findings provide a more comprehensive understanding of the pathological mechanisms underlying vascular thrombus formation in HCC and may inform the development of predictive and therapeutic strategies targeting MVI and PVTT.

## Results

### TF is upregulated in HCC tissues and is associated with poor prognosis

**Fig. 1:**
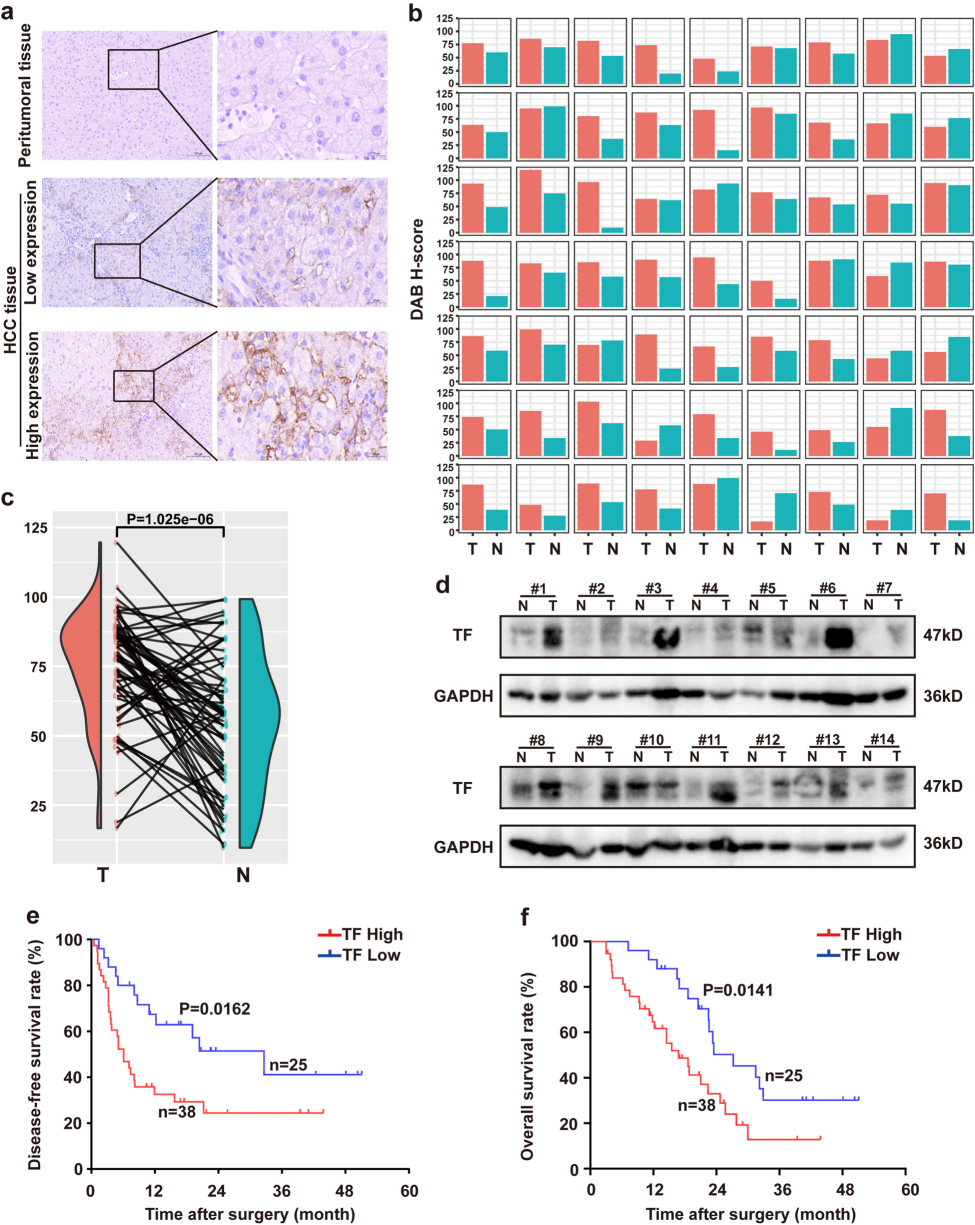
TF expression is elevated in HCC tissues and correlates with poor prognosis and survival. **a** Representative immunohistochemical (IHC) staining of TF protein expression in HCC tissue sections (*n* = 63). Scale bar = 20 μm/100 μm. **b** Quantitative analysis of IHC staining using the DAB H‑score. **c** Distribution of DAB H‑scores visualized by violin plots. **d** Western blot analysis of TF expression in fresh HCC tumor tissues (T) and matched adjacent non‑tumor tissues (N) (*n* = 14); GAPDH served as the loading control (**e**–**f**) Kaplan–Meier survival analysis demonstrating the association between TF expression and overall survival in HCC patients

Paired cancerous and adjacent normal tissue samples were collected from 63 patients with pathologically confirmed HCC. TF protein expression was evaluated by immunohistochemical staining. Variable degrees of positive TF expression were observed in HCC tissues, whereas expression levels were predominantly lower in the corresponding adjacent normal tissues (Fig. 1a). Using the HALO Multiplex IHC v3.0 (Indica Labs; Albuquerque, NM, USA) algorithm, 3,3′-diaminobenzidine staining was quantitatively identified and statistically analyzed for each sample. Statistical analysis confirmed that TF expression was significantly higher in HCC tissues than in paired adjacent normal liver tissues (Fig. 1b and c, *P* = 1.025e-06). To further validate these findings, TF protein expression was examined using western blot analysis in 14 paired surgically resected HCC and adjacent normal tissues. Consistently, TF protein levels were significantly elevated in HCC tissues compared with matched normal tissues (Fig. 1d).

Integration of TF expression data with clinicopathological characteristics and follow-up information from 63 patients with HCC, revealed that high TF expression was significantly associated with vascular invasion (*P* = 0.014), lymphatic metastasis (*P* = 0.029), and advanced TNM stage (*P* = 0.011) (Supplementary Table 1). Survival analysis demonstrated that patients with high TF expression had significantly shorter disease-free survival (DFS; *P* = 0.0162) and overall survival (OS; *P* = 0.0141) than those with low TF expression (Fig. 1e, f). These findings were further validated using independent public datasets from TCGA-LIHC, in which patients classified as high TF risk by our model consistently exhibited significantly poorer OS than those in the low-risk group (*P* = 0.0094) (Fig. S1). Collectively, these results indicate that TF is frequently upregulated in HCC and is closely associated with adverse pathological features, including vascular invasion, lymphatic metastasis, and reduced DFS and OS.

### TF significantly promotes the proliferation, migration and invasion of HCC cells in vitro

**Fig. 2:**
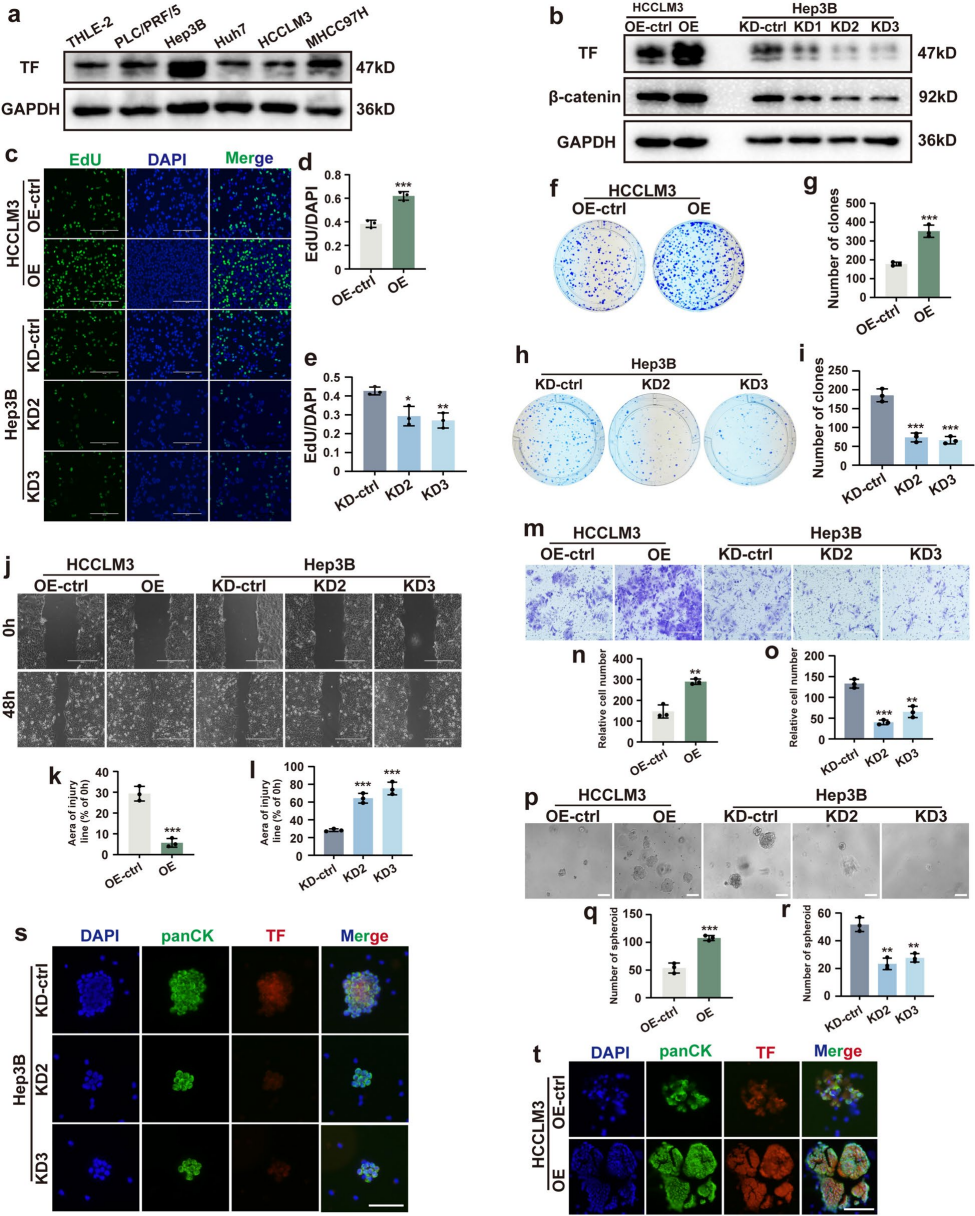
TF facilitates the proliferation, migration, and spheroid formation ability of HCC cells in vitro. **a** Western blot analysis of TF expression in a normal liver cell line and various hepatocellular carcinoma (HCC) cell lines. **b** Western blot detection of TF expression following overexpression or knockdown, along with β-catenin expression. **c**-**e** Cell proliferation potential of HCC cells upon TF up- or down-regulation was evaluated using EdU assay. **f**-**i** Clonogenic ability of HCC cells was assessed by plate colony formation assay. **j**-**l** Cell migration was examined by wound-healing assay; wound closure was measured at 0 h and 48 h post-scratch. **m**-**o** Representative images and quantitative analysis of transwell assays demonstrating the invasive capability of HCC cells. **p**-**r** Three-dimensional spheroid formation assay was used to evaluate spheroidizing ability of HCC cells (scale bar = 100 μm). **s** and **t** Cellular immunofluorescence staining of TF and panCK (epithelial marker) in HCC cell spheroids (scale bar = 100 μm). ^*^P < 0.05, ^**^P < 0.01, ^***^P < 0.001

TF protein expression was next assessed in five HCC cell lines (PLC/PRF/5, Hep3B, Huh7, HCCLM3 and MHCC97H) and the normal liver cell line (THLE-2). TF expression was generally higher in HCC cell lines than in normal liver cells (Fig. 2a). Based on relative TF expression levels, Hep3B cells (highest TF expression) and HCCLM3 cells, (lowest TF expression, abbreviated as LM3) were selected for subsequent experiments.

To investigate the functional role of TF on the biological functions of HCC cells, lentiviral vectors were constructed to induce TF overexpression or knockdown. These vectors were packaged into lentiviruses and used to infect HCCLM3 cells (for TF overexpression) and Hep3B cells (for TF knockdown). Following puromycin selection, stable cell lines were successfully established (Fig. S2). Western blot analysis confirmed robust TF overexpression (OE). Among three knockdown constructs, vectors 2 and 3 exhibited the highest silencing efficiency and were selected for further experiments (designated KD2 and KD3) (Fig. 2b).

The effects of TF expression on HCC cell proliferation were evaluated using EdU incorporation and colony formation assays. TF overexpression significantly enhanced the proliferative capacity of HCC cells compared with control, whereas TF knockdown markedly suppressed proliferation in Hep3B cells (Fig. 2c-e). Consistently, colony formation assay revealed a significant increase in colony number in TF-overexpressing cells and a pronounced reduction in colonies following TF-knockdown (Fig. 2f-i).

The role of TF in HCC cell migration and invasion was further examined using wound-healing and transwell assays. TF overexpression substantially promoted both migratory and invasive capacities of HCC cells relative to controls, whereas TF knockdown significantly impaired these processes (Fig. 2j-o). Collectively, these in vitro experiments demonstrate that TF plays a critical role in promoting HCC cell proliferation, migration, and invasion.

### TF facilitates HCC cell spheroid formation in vitro

A three-dimensional (3D) cell spheroid formation assay was performed to evaluate the spheroid-forming capacity of HCC cells in vitro. The 3D spheroid model more closely mimics in vivo tumor microenvironment and enables evaluation of tumor cell survival under mechanical stresses, such as circulatory shear forces. TF-overexpressing HCC cells exhibited a significantly enhanced, spheroid-forming ability, characterized by increased spheroid number and size compared with the control group. Conversely, TF knockdown markedly inhibited in vitro spheroid formation (Fig. 2p-r). Subsequently, spheroids were digested, resuspended, and used to prepare cell smears. Immunofluorescence staining for pan-cytokeratin (panCK) and TF confirmed that TF overexpression enhanced spheroid formation, whereas TF knockdown significantly impaired this capacity (Fig. 2s and t). Collectively, these findings suggest that elevated TF expression in the tumor microenvironment may promote the spheroid-forming capacity of CTCs released from primary HCC tumor during hematogenous dissemination, thereby improving their survival in peripheral circulation and facilitating metastatic colonization.

### TF drives tumorigenesis and metastatic progression of HCC in vivo

**Fig. 3:**
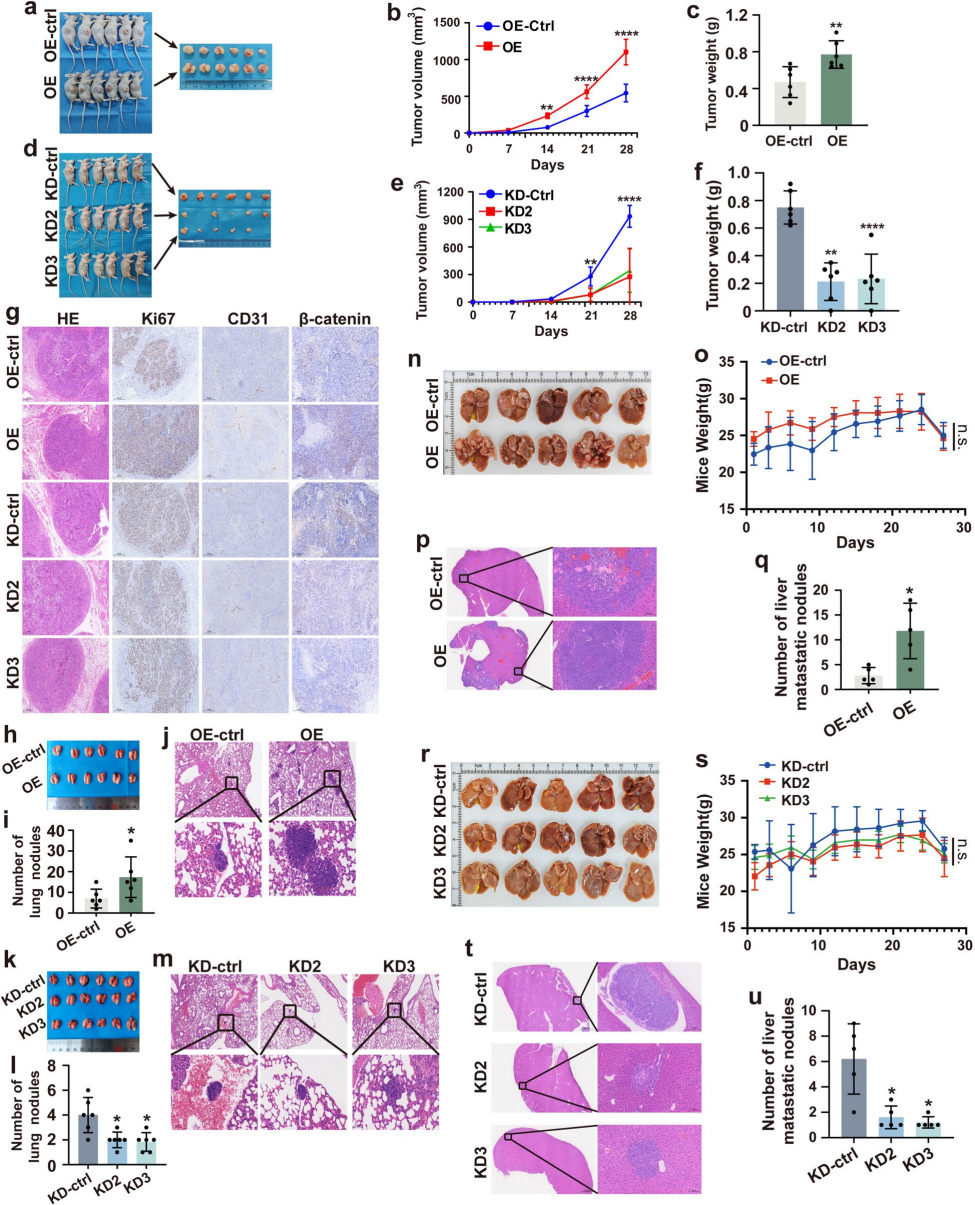
In vivo assessment of TF modulation on HCC tumor growth and metastasis. **a** and **d** A nude mouse xenograft model were established to assess the effect of TF upregulation or downregulation on hepatocellular carcinoma (HCC) tumor growth *in vivo* (*n* = 6 mice per group). **b**, **c** and **e**, **f** Tumor volume and weight were measured at regular intervals. **g** Representative immunohistochemical staining of tumor tissues shows the expression of Ki67, CD31, and β-catenin across experimental groups. Scale bar = 200 μm. **h** and **k** A pulmonary metastasis model was generated by tail vein injection. Gross images of lungs are shown for each group (*n* = 6 mice per group). **j** and **m** Representative hematoxylin and eosin (HE)-stained sections illustrate pulmonary metastatic nodules. Scale bar = 50 μm/500 μm. **i** and **l** The number of pulmonary metastatic nodules was quantified for each group. **n** and **r** A liver metastasis model was established via intrasplenic injection. Gross images of livers are presented for each group (*n* = 5 mice per group). **o** and **s** Mouse body weight was monitored regularly throughout the experiment. **p** and **t** Representative HE-stained sections depict liver metastatic nodules. Scale bar = 100 μm/2000 μm. **q** and **u** The number of liver metastatic nodules was statistically analyzed for each group. ^*^P < 0.05, ^**^P < 0.01, ^****^P < 0.0001, n.s., no significance

To evaluate the effect of TF on HCC cells proliferation in vivo, a subcutaneous xenograft tumor model was established in nude mice. Mice implanted with TF-OE stable cells (*n* = 6) exhibited enhanced proliferation and tumorigenicity in vivo, forming tumors with significantly larger volumes and higher weights compared with those in the control group (*n* = 6). In contrast, TF-KD HCC cells (*n* = 6) exhibited markedly suppressed tumor growth in vivo (Fig. 3a-f). Immunohistochemical analysis of xenograft tumor tissues demonstrated that TF overexpression was associated with increased expression of Ki67, CD31, and β-catenin, indicating enhanced tumor cell proliferation, neovascularization, and activation of oncogenic signaling. Conversely, TF knockdown resulted in reduced Ki67-positive cells, decreased CD31 expression, and lower β-catenin protein levels (Fig. 3g).

To validate the effect of TF on intrahepatic and extrahepatic metastasis in vivo, we established lung and intrahepatic metastasis models in nude mice by injecting TF-OE or TF-KD stable cell lines via tail vein and splenic routes, respectively. Four weeks after injection, mice in the TF-OE group exhibited significantly higher numbers of hepatic and pulmonary metastatic nodules than those in the control group, whereas TF knockdown markedly reduced the metastatic burden (Fig. 3h-u). These findings indicate that loss of TF expression effectively suppresses both intrahepatic and extrahepatic metastasis of HCC cells in vivo (Fig. 3h-m). No significant adverse effects were observed during the animal experiments.

### Elevated TF expression enhances dissemination of CTCs/CTMs in the peripheral circulation, and promotes hematogenous metastasis in HCC cells

HCC, is a highly vascular malignancy frequently characterized by vascular invasion or tumor thrombus formation, which contributes to intrahepatic/and extrahepatic metastasis and is associated with poor prognosis.

**Fig. 4:**
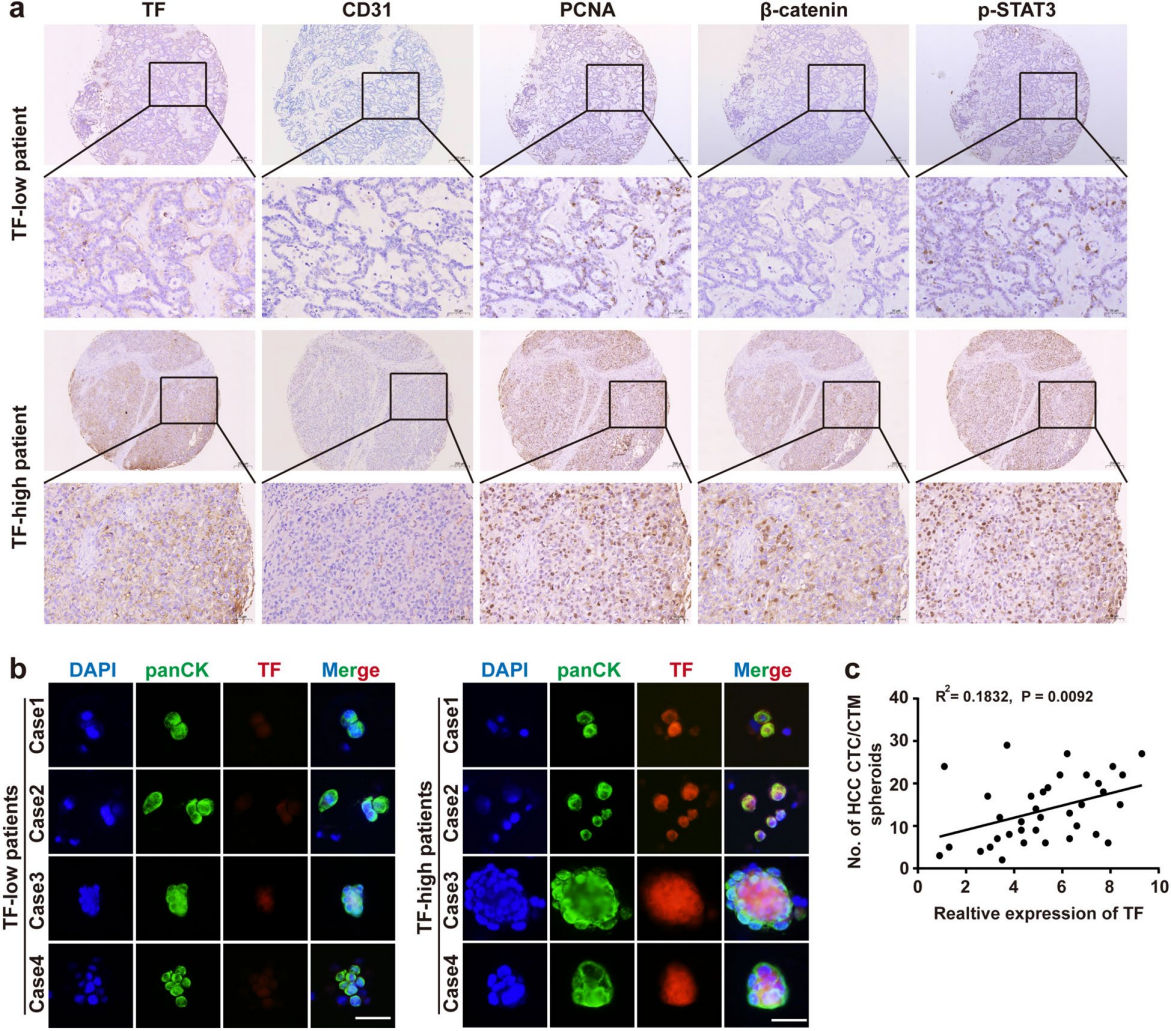
Elevated TF expression promotes angiogenesis and facilitates tumor cell survival and dissemination in the circulatory system, ultimately driving microvascular invasion. **a** Representative immunohistochemical staining of serial tissue sections from HCC patients, showing expression patterns of TF, CD31, PCNA, β-catenin, and p-STAT3. Scale bar = 50 μm/200 μm. **b** Cellular immunofluorescence staining of TF and panCK (an epithelial marker) in CTCs and CTMs isolated from peripheral blood of HCC patients. Scale bar = 100 μm. **c** Correlation between relative TF expression levels and CTC/CTM enumeration in the same cohort of HCC patients (*n* = 36)

To investigate the clinical relevance of TF in this phase context, surgically resected tumor tissues and matched peripheral blood samples were collected from 36 patients with HCC. Immunohistochemical analysis of serial tissue sections revealed that tumors with high TF expression also exhibited elevated levels of proliferating cell nuclear antigen (PCNA), CD31, β-catenin, and phosphorylated STAT3 (Fig. 4a), consistent with our in vivo findings (Fig. 3g). Collectively, these data suggest that elevated TF expression is associated with enhanced tumor cell proliferation, increased neovascularization, and activation of oncogenic signaling pathways.

Subsequently, CTCs/CTMs were isolated from the peripheral blood samples of matched patient with HCC and quantified by immunofluorescence. Patients with high TF expression exhibited significantly higher numbers of CTCs/CTMs, along with larger CTM spheroids characterized by more compact and cellular architecture. In contrast, patients with low TF expression showed fewer CTCs/CTMs, smaller CTM volumes, and looser spheroidal structures (Fig. 4b). Correlation analysis further revealed a positive association between TF expression levels and CTC/CTM counts in the peripheral blood of patients with HCC (Fig. 4c).

### STAT3 directly regulates TF expression and combines with β-catenin in its intracellular signaling cascade

**Fig. 5:**
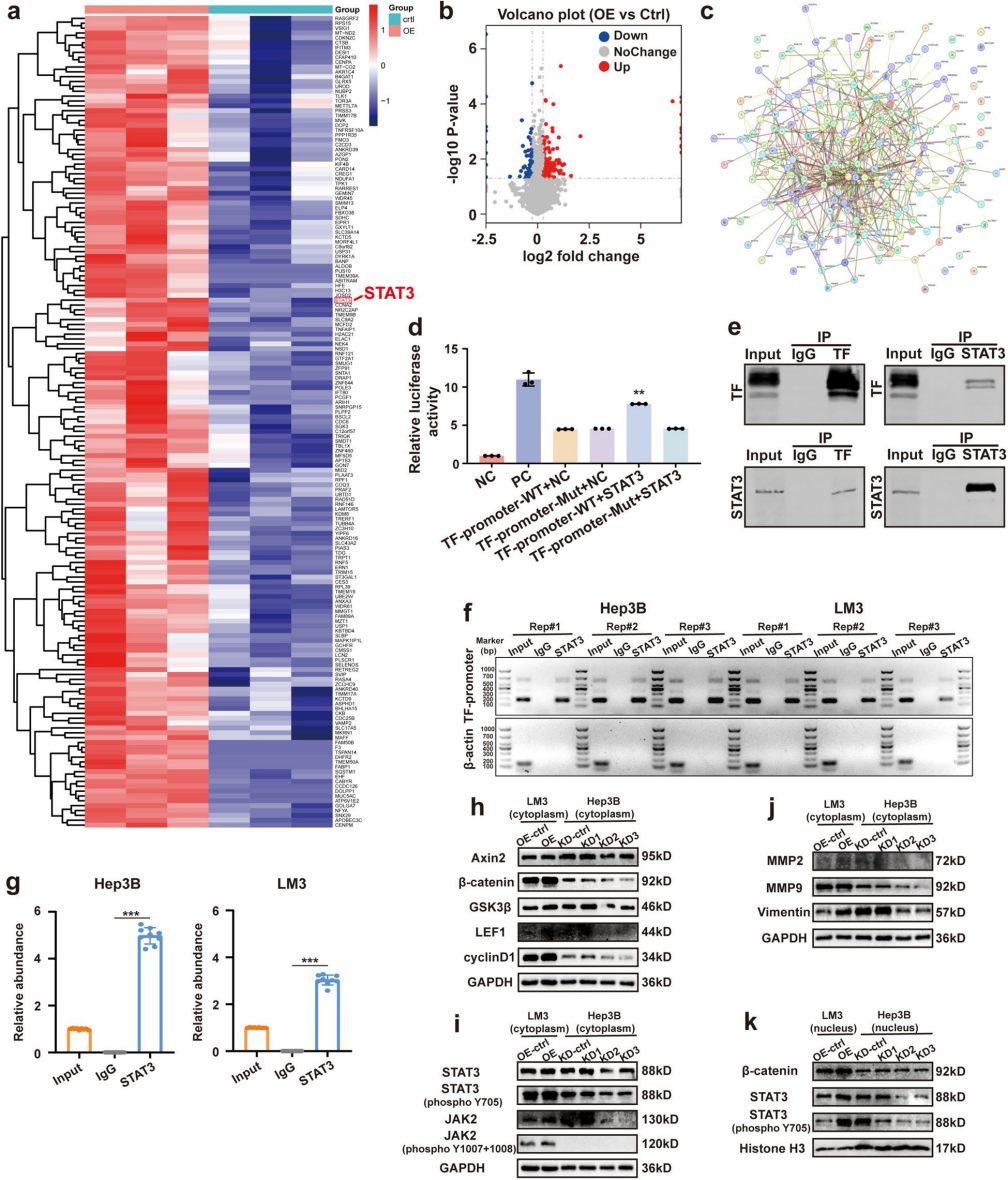
TF may drive tumorigenesis through a positive feedback loop involving STAT3 and β-catenin regulation. **a** Heatmap illustrating differentially expressed proteins identified by cluster analysis between the Ctrl and TF-OE groups. **b** Volcano plot showing the distribution of differentially expressed proteins (red: upregulated; blue: downregulated; gray: nonsignificant). **c** Protein-protein interaction (PPI) network constructed using STRING. **d** Dual-luciferase reporter assay confirming direct binding of TF to the STAT3 promoter. ^**^P < 0.01 vs TF-promoter-mut + STAT3 group. **d** Co-immunoprecipitation (Co-IP) demonstrating endogenous interaction between TF and STAT3 in HCCLM3 cells. **f** and **g** Chromatin immunoprecipitation (ChIP) assays in Hep3B and HCCLM3 cells. Enrichment of the TF promoter region was quantified by qPCR. Data represent mean ± SD from three independent ChIP experiments. ^***^P < 0.001compared to IgG group. **h** and **i** Western blot analysis of Wnt/β-catenin and JAK2/STAT3 signaling pathway proteins in HCC cells with stable TF overexpression or knockdown. GAPDH served as loading control. **j** Western blot detection of metastasis-associated proteins (MMP2, MMP9, Vimentin) in the cytoplasm of HCC cells. **k** Nuclear protein levels of β-catenin, STAT3, and phosphor-STAT3 measured by western blot

To further elucidate the molecular mechanisms by which TF promotes HCC progression, we performed data-independent acquisition mass spectrometry in TF-overexpressing HCC cells to identify potential interacting proteins. MS and subsequent bioinformatics analyses, including protein-protein interaction (PPI) network construction, revealed that among the upregulated differentially expressed proteins, the transcription factor STAT3, known to play a critical role in tumor progression, was identified as a potential TF-interacting protein (Fig. 5a-c). Gene Ontology (GO) and KEGG pathway analyses further indicated that TF overexpression was closely associated with biological processes related to cellular metabolic signaling and apoptosis (Fig. S3). We next investigated whether STAT3 directly regulates TF expression. Dual-luciferase reporter assays demonstrated that co-transfection with STAT3 significantly enhanced TF promoter activity, indicating that STAT3 promotes TF transcription (Fig. 5d). Furthermore, co-immunoprecipitation (co-IP) combined with western blotting confirmed a direct interaction between TF and STAT3 in HCC cells (Fig. 5e). Chromatin immunoprecipitation followed by quantitative polymerase chain reaction (ChIP-qPCR) further revealed significant enrichment of STAT3 at the TF promoter region, providing direct molecular evidence that STAT3 activates TF transcription (Fig. 5f and g).

Previous studies have shown that TF mediates intracellular signaling through activation of PARs. In HCC cells, PAR1 and PAR2 are the predominant PAR subtypes expressed on the cell surface. Western blot analysis demonstrated that TF overexpression selectively increased PAR1 protein levels, suggesting that TF primarily signals through PAR1 to initiate downstream intracellular signaling (Fig. S4a). Consistently, pharmacological inhibition of PAR1 with the specific antagonist Vorapaxar significantly attenuated malignant biological phenotypes in TF-overexpressing HCC cells (Fig. S4b-4i). To further delineate the downstream signaling mechanisms, we examined the expression, phosphorylation, and subcellular localization of β-catenin and STAT3 in TF-overexpressing HCC cells following PAR1 inhibition. Immunofluorescence analysis corroborated these findings by showing reduced nuclear accumulation of β-catenin and p-STAT3 in Vorapaxar-treated cells (Fig. S4j). Vorapaxar treatment markedly reduced the protein levels of β-catenin, STAT3, and p-STAT3 in both cytoplasmic and nuclear fractions (Fig. S4k). Collectively, these results indicate that TF initiates intracellular signaling primarily through PAR1 activation, thereby driving malignant phenotypic changes and downstream signal transduction.

The canonical Wnt/β-catenin signaling pathway plays a pivotal role in HCC development. As a core component of this pathway, β-catenin is frequently overexpressed in HCC, leading to activation of downstream effector genes, such as GSK3β and c-Jun, thereby promoting tumor progression. In this study, TF overexpression significantly increased β-catenin expression, along with its downstream effectors, Axin2, GSK3β, LEF1, and cyclinD1, whereas TF knockdown produced the opposite effects (Figs. 2b and 5h). In parallel, TF overexpression markedly elevated levels of p-STAT3 (Y705), while TF knockdown substantially reduced the expression of STAT3, p-STAT3 (Y705), and JAK2 (Fig. 5i). Nuclear protein extraction and western blot analysis further demonstrated that TF overexpression promoted nuclear accumulation of β-catenin, STAT3, and p-STAT3 (Y705) (Fig. 5k). These findings support a dual regulatory mechanism in which TF overexpression enhances nuclear translocation of these oncogenic transcription factors, thereby sustaining malignant phenotypes in HCC cells. Concurrently, aberrant nuclear accumulation of these factors likely activates the TF promoter, further increasing TF transcription and protein expression. Together, these processes establish a positive feedback loop that perpetuates and amplifies oncogenic signaling in HCC.

In addition, TF overexpression of significantly upregulated the expression of MMP2, MMP9, and Vimentin, whereas TF knockdown of TF reduced their expression (Fig. 5j). These results suggest that elevated TF levels enhance HCC cell migratory capacity and promote extracellular matrix (ECM) degradation, thereby facilitating invasion and metastasis. In contrast, examination of key components of the AKT signaling pathway revealed no substantial changes in protein expression following TF modulation (Fig. S5).

Collectively, these results suggest that TF initiates intracellular signaling through PAR1 activation on the HCC cell surface. TF subsequently enhances the expression and nuclear accumulation of β-catenin and STAT3, activating the Wnt/β-catenin and JAK2/STAT3 pathways., In turn, nuclear STAT3 directly promotes TF transcription, forming a positive feedback loop that sustains oncogenic signaling. Furthermore, TF overexpression enhances cell motility and ECM degradation, thereby promoting metastatic dissemination.

### Combined TF deprivation and β-catenin and STAT3 inhibition markedly suppress HCC cells proliferation and migration in vitro

**Fig. 6:**
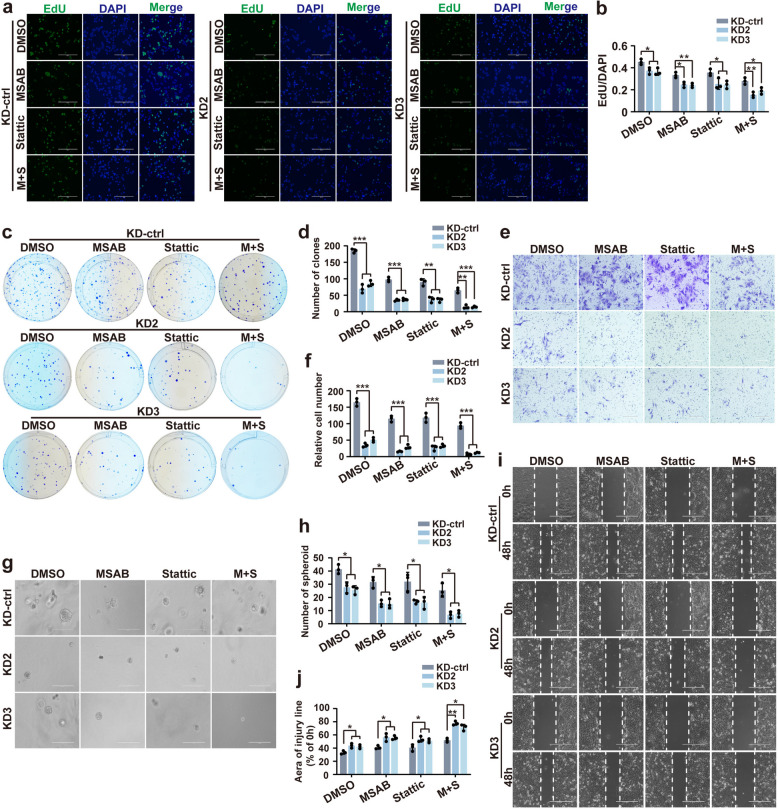
Knockdown of TF combined with inhibition of β-catenin and STAT3 signaling significantly suppresses the proliferation and metastatic potential of HCC cells in vitro. **a** Representative images and **b** quantitative analysis of EdU assay demonstrating the proliferation capacity of HCC cells across treatment groups. **c** Colony formation assay showing clonogenic potential and **d** corresponding quantification in different treatment groups. **e** Representative images and **f** quantitative results of transwell assays reflecting the invasive ability of HCC cells under each condition. **g** Three-dimensional spheroid formation assay illustrating spheroid growth and **h** its quantification across groups (scale bar = 100 μm). **i** Wound-healing assay images captured at 0 h and 48 h post-scratch, along with (**j**) statistical analysis of wound closure rates, indicating migratory capacity. ^*^P < 0.05, ^**^P < 0.01, ^***^P < 0.001

To investigate the combined effects of TF deprivation and inhibition of β-catenin/and STAT3 signaling, stable TF-KD HCC cell lines were treated with the β-catenin inhibitor MSAB and the STAT3 inhibitor Stattic in in vitro functional assays, demonstrated that TF knockdown combined with dual pathway inhibition markedly suppressed HCC cell proliferation, colony formation, migration, invasion, and spheroid-forming capacity to a greater extent than either control or single agent treatments (Fig. 6).

In TF-overexpressing HCC cells, treatment with β‑catenin and STAT3 signaling pathway inhibitors, either alone or in combination, was followed by western blot analysis of downstream markers associated with ECM degradation and metastasis, such as MMP2, MMP9, Vimentin, E-cadherin, N-cadherin, and Snail. Dual pathway inhibition more effectively suppressed the expression of pro-invasive and ECM-degrading markers than single-agent inhibition (Fig. S6).

To determine whether crosstalk or synergy exists between the β-catenin and STAT3 signaling pathways, pathway-specific agonists and co-immunoprecipitation assays were employed. Activation of β-catenin signaling with the agonist SKL2001 did not alter STAT3 expression or phosphorylation in either the cytoplasmic or nuclear compartments. Similarly, STAT3 activation using the agonist ML115 did not affect β-catenin expression (Fig. S7a-7b). Co-immunoprecipitation experiments further confirmed the absence of endogenous PPI between β-catenin and STAT3 or between β-catenin and p-STAT3 in TF-OE cells (Fig. S7c).

Collectively, these findings indicate that β-catenin and STAT3 function as independent downstream effectors of TF signaling. The pronounced inhibitory effects observed following dual pathway blockade are therefore attributable to additive, rather than synergistic effects.

### Combined TF suppression and β-catenin/STAT3 inhibition markedly inhibit HCC tumorigenesis and metastasis in vivo

**Fig. 7:**
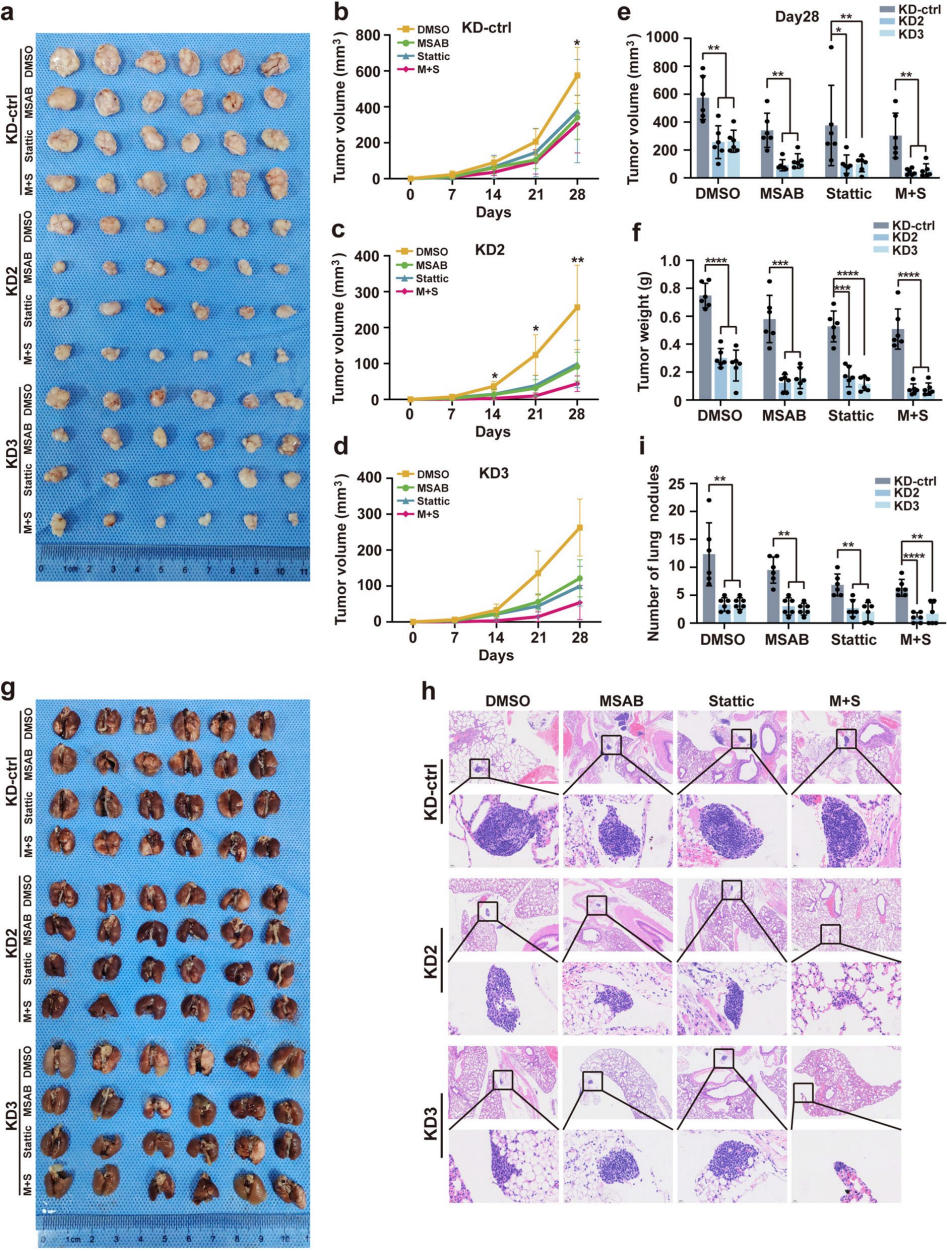
Suppression of TF expression together with β-catenin and STAT3 inhibitors potently inhibits HCC proliferation and metastasis in vivo. **a** A xenograft model was established in nude mice to assess the effects of β-catenin and STAT3 inhibitors, administered individually or in combination, on the tumor growth of HCC cells (TF-KD) in vivo (*n* = 6 mice per group). **b**-**f** Tumor volume and weight were measured periodically across all groups. **g** A pulmonary metastasis model was generated via tail vein injection. Lung specimens from each group (*n* = 6 mice per group) are shown. **h** Representative hematoxylin and eosin (HE)-stained sections illustrating pulmonary metastatic nodules in each group. Scale bar = 20 μm/200 μm. **i** Quantitative analysis of pulmonary metastatic nodules in each group. ^*^P < 0.05, ^**^P < 0.01, ^***^P < 0.001, ^****^P < 0.0001

To further investigate the combined effect of TF knockdown and dual inhibitor treatment on the tumorigenesis and metastasis of HCC cells in vivo, we established two murine models: a subcutaneous xenograft model and a tail vein injection-induced lung metastasis model. Combined TF knockdown and dual inhibition of β-catenin and STAT3 significantly suppressed tumor growth and lung metastatic burden in HCC cells compared with control or single-treatment groups (Fig. 7). No significant adverse effects were observed during the experimental period. Overall, these results demonstrate that targeting TF in combination with β-catenin and STAT3 inhibitor substantially inhibits HCC tumorigenesis and metastatic progression both in vitro and in vivo.

## Discussion

In recent years, the clinical management of HCC has expanded beyond surgical resection to include various treatment modalities such as local ablation, interventional therapy, radiotherapy, chemotherapy, and immunotherapy. Despite these advances, postoperative survival outcomes for patients with HCC remain unsatisfactory [17]. Current therapeutic challenges largely stem from the intrinsic biological aggressiveness of HCC, limitations of available therapies, and substantial interpatient heterogeneity. Among the most aggressive phenotypic features of HCC is vascular tumor thrombus formation or vascular invasion, which frequently leads to intrahepatic recurrence and distant metastasis, thereby markedly worsening patient prognosis [18]. Although the prognostic significance of vascular invasion in HCC is well established, its underlying etiology and molecular mechanisms remain incompletely understood.

In this study, we identified TF as a key regulator of vascular invasion of in HCC and elucidated the molecular mechanism underlying its pro-metastatic effects. Vascular invasion begins with the detachment of tumor cells from the primary lesion and their entry into the bloodstream, leading to the formation of CTCs. Individual CTCs are highly vulnerable to mechanical shear stress and immune surveillance within the bloodstream, and only a small fraction survive to seed metastatic lesions. These surviving cells may exist as single CTCs or aggregate to form CTMs, which can comprise two or more (and in some cases up to ≥ 50) other CTCs [19, 20]. Previous studies have demonstrated that CTMs possess superior survival capacity, enhanced metastatic potential, and increased resistance to chemotherapy, thereby contributing to poor clinical outcomes.

Among coagulation factors, TF is unique as the only type I transmembrane protein expressed on the cell surface. As the receptor for coagulation factor VII, TF binds and activates factor VII, thereby initiating the extrinsic coagulation cascade [21]. TF is highly expressed in various malignancies, including HCC [11, 14, 22, 23], and CTCs derived from primary tumors similarly express TF on their surface. Upon exposure to peripheral blood, TF-positive CTCs can activate coagulation factor VII, triggering the extrinsic coagulation pathway, ultimately promoting the formation of CTM. Consistent with this mechanism, our results demonstrate that TF overexpression significantly enhances the spheroid-forming capacity of HCC cells in vitro, whereas TF knockdown markedly suppresses this potential (Fig. 2s and t). Clinically, patients with HCC exhibiting high TF expression not only show significantly increased numbers of CTCs/CTMs in peripheral blood but also larger, more compact, and denser CTM spheroids. These features likely confer a survival advantage under circulatory stress, including hemodynamic shear forces and immune-mediated clearance, thereby facilitating hematogenous metastasis through vascular invasion.

**Fig. 8:**
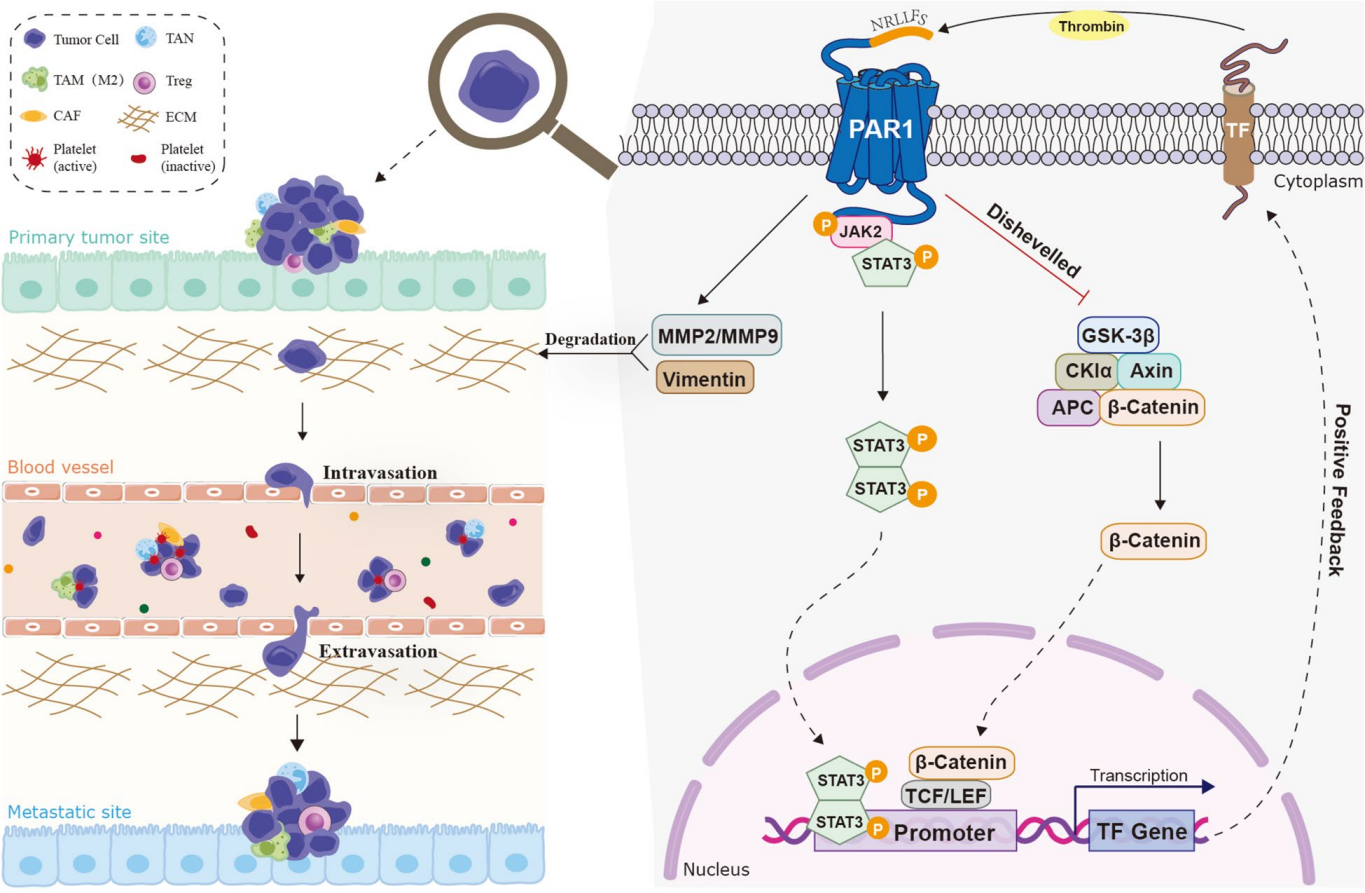
Schematic of the underlying mechanism of TF in HCC. TF is overexpressed in HCC tissues. TF activates PAR1 on the surface of HCC cells, leading to the transduction of the Wnt/β-catenin and JAK2/STAT3 signaling pathways. This activation, on one hand, modulates malignant phenotypes including cell proliferation, growth, metastasis, in vitro spheroid formation, angiogenesis, and hematogenous dissemination-the latter by enhancing the survival and spread of CTCs/CTMs. On the other hand, TF upregulates MMP2 and MMP9, which degrade the ECM to facilitate HCC cell migration and colonization, as well as Vimentin, a marker linked to metastatic behavior. Furthermore, TF promotes nuclear translocation and elevated expression of STAT3 and β-catenin. Notably, nuclear STAT3 binds directly to the TF promoter, creating a positive feedback loop that further amplifies TF expression. In summary, TF overexpression in HCC enhances tumor cell proliferation, metastatic potential, angiogenesis, hematogenous survival and dissemination, and expression of ECM-degrading genes. Collectively, these mechanisms promote HCC invasion across microvessels and major blood vessels, ultimately driving intrahepatic and extrahepatic metastasis

A central objective of this study was to elucidate the molecular mechanisms by which TF promotes vascular invasion in HCC. Previous studies have shown that membrane bound TF can mediate intracellular signaling through activation of PARs, thereby enhancing malignant phenotypes, including proliferation, migration, resistance to apoptosis, and tumor angiogenesis, via the induction of pro-angiogenic factors and other cytokines [24]. Our findings demonstrate that TF overexpression selectively upregulates PAR1 protein expression, suggesting that TF primarily activates PAR1 to initiate downstream signaling in HCC cells. Pharmacological inhibition of PAR1 significantly attenuated TF-induced malignant phenotypes, underscoring its functional importance. The canonical Wnt/β-catenin signaling pathway is critically involved in HCC pathogenesis [25]. As a core component of this pathway, β-catenin is frequently overexpressed in HCC, leading to activation of downstream effectors, such as GSK3β and c-Jun and driving tumor progression. Activation of Wnt signaling stabilizes β-catenin, allowing its nuclear translocation and interaction with TCF/LEF transcription factors to induce pro-oncogenic targets (e.g., c-Myc, Cyclin D1, Snail, Twist, MMPs). These events promote tumor cell proliferation, epithelial-mesenchymal transition, and ECM-degradation via matrix metalloproteinases, ultimately facilitating metastasis [26]. In the present study, TF overexpression markedly increased the expression and nuclear accumulation of β-catenin in the Wnt/β-catenin pathway, as well as STAT3 in the JAK2/STAT3 pathway. Nuclear β-catenin sustained transcription of oncogenic targets, thereby reinforcing malignant phenotypes in HCC cells. Concurrently, nuclear accumulation of STAT3/p-STAT3 enhances TF promoter activity, establishing a positive feedback loop that perpetuates cascade signaling activation. Together, these findings provide an integrated mechanistic overview, as summarized in Fig. 8.

Even after radical resection, the 5-year recurrence rate in patients with HCC and vascular invasion remains as high as 70%, largely due to preoperatively undetectable micrometastases or postoperative proliferation of residual cancer cells [27]. Current therapeutic options for HCC with vascular invasion include targeted therapies (e.g., sorafenib, lenvatinib), transarterial chemoembolization (TACE), radioactive particle implantation (e.g., Yttrium-90, Iodine-125), and immunotherapy (e.g., nivolumab). However, the efficacy of targeted agents is often compromised by tumor heterogeneity and the development of drug resistance, resulting in limited benefit for a subset of patients [28]. Conventional TACE efficacy may be reduced by heterogeneous tumor vascularization, including extrahepatic collateral circulation, and repeated procedures can exacerbate liver injury, particularly in patients with underlying cirrhosis [29]. Radioactive particle implantation requires high technical precision, and factors such as respiratory motion or unfavorable tumor location may increase procedural risks [30]. Although immunotherapy shows promise in HCC with vascular invasion, it may also exacerbate liver dysfunction, promote a pro-tumor immune microenvironment, or induce immune-related adverse events [31]. Currently, no unified consensus exists regarding immunotherapy in this patient population, underscoring the need for further clinical investigation. Consequently, optimal management of HCC with vascular invasion requires a multidisciplinary approach that integrates local and systemic therapies. Nevertheless, controlling recurrence, overcoming drug resistance, and preserving liver function continue to pose significant challenges, highlighting the need for more prospective studies to refine and optimize therapeutic strategies.

In recent years, TF has emerged as a molecule of interest in HCC with vascular invasion due to its pro-coagulant and pro-tumorigenic functions and its potential as a therapeutic target. Prior studies have demonstrated that TF activates downstream signaling pathways, such as MAPK/ERK and PI3K/AKT, via PAR2, thereby promoting tumor invasion and angiogenesis [20]. Our findings extend this knowledge by demonstrating that PAR1 is preferentially activated in HCC cells with high TF expression, facilitating intracellular signal transduction. Under conditions of TF overexpression, key effector molecules of the Wnt/β-catenin and JAK2/STAT3 pathways, β-catenin and STAT3, show markedly increased nuclear accumulation. Furthermore, STAT3 directly enhances TF transcription by activating its promoter, forming a positive feedback loop that amplifies TF-mediated signaling. Notably, combined suppression of TF and inhibition of β-catenin and STAT3 pathway inhibitors produced significant inhibitory effects on HCC cell proliferation and metastasis both in vitro and in vivo, suggesting a promising therapeutic strategy for metastatic HCC.

Several limitations of this study should be acknowledged. First, the analyses were primarily based on samples from a single center with a relatively limited cohort size, which may restrict the generalizability of the findings. Future studies incorporating larger, multicenter cohorts are warranted to validate these results. Second, further investigations are required to confirm the long-term efficacy and potential adverse effects of TF-targeted therapies in metastatic HCC. In addition, the development of antibody-based therapeutics, TF-targeting conjugated drugs, and their synergistic effects in combination with immunotherapy, warrant additional experimental and clinical validation. Addressing these issues will contribute to a more comprehensive understanding of TF biology and its therapeutic potential in HCC.

## Conclusions

In summary, our study demonstrates that TF plays a critical role in HCC vascular invasion and metastasis by activating the TF-PAR1/Wnt/β-catenin and JAK2/STAT3 signaling pathways, thereby establishing a positive feedback loop that sustains oncogenic signaling. These findings suggest that combined targeting of TF and the Wnt/β-catenin and JAK2/STAT3 pathways may represent a promising adjunctive therapeutic strategy for HCC.

## Materials and methods

### Tissue samples and cell lines

This study included surgically resected paired HCC and adjacent normal tissue samples (*n* = 14) for western blot analysis. These specimens were obtained from the Eastern Hepatobiliary Surgery Hospital, Navy Military Medical University between March and July 2022. Additionally, 63 pairs of paraffin-embedded HCC tissues collected between January and March 2020 at the same institution were used for immunohistochemical staining. The final follow-up date was April 30, 2025. During follow-up, two patients (3.17%) were lost to contact, primarily due to changes in contact information and relocation, these patients were censored at their last documented clinical visit in the survival analyses. All postoperative pathological diagnoses were confirmed. Surgical specimens together with matched peripheral blood samples from 36 patients with HCC (for CTC/CTM detection) were also obtained from the Eastern Hepatobiliary Surgery Hospital between March and September 2022. The use of human tissue samples, blood samples, and clinical data was approved by the Ethics Committee of the Eastern Hepatobiliary Surgery Hospital, Navy Military Medical University (Approval number: EHBHKY2020-01-003, Shanghai, China). Written informed consent was obtained from all participants.

Human HCC cell lines (Huh7, Hep3B, PLC/PRF/5, HCCLM3, and MHCC97H) and the immortalized normal liver cell line THLE-2 were obtained from the Chinese Academy of Sciences Cell Bank (Shanghai, China). All cell lines were authenticated by short tandem repeat profiling and maintained according to the supplier’s protocols. Cells were cultured in Dulbecco's Modified Eagle Medium (DMEM) supplemented with 10% fetal bovine serum (FBS; Gibco, USA), 100 units/mL penicillin, and 100 µg/mL streptomycin (Invitrogen, USA) at 37 °C in a humidified incubator with 5% CO₂.

### Co-IP assay

Co-IP assays were conducted to validate the interactions of TF with STAT3 as well as β-catenin with STAT3/p-STAT3. HCC cells overexpressing TF (TF-OE) or carrying the control vector were lysed using IP lysis buffer (Beyotime, China). The resulting lysates were separately incubated overnight at 4 °C with antibodies against TF, β-catenin, STAT3, and p-STAT3. Subsequently, Protein A/G-agarose beads were introduced for 2 h. Following incubation, the beads were collected and washed three times with lysis buffer. Bound proteins were eluted, denatured in 5 × sodium dodecyl sulphate (SDS) loading buffer, and subjected to western blot analysis. Details regarding the antibodies used are provided in Supplementary Table 4.

### Chromatin immunoprecipitation (ChIP)

ChIP assay was performed to examine the interaction between STAT3 and the TF promoter region. Briefly, cells were washed twice with phosphate-buffered saline and fixed with 1% formaldehyde for 10 min at room temperature. The cross-linking reaction was quenched with 0.125 M glycine. After harvesting, cells were resuspended in SDS buffer (50 mM Tris-HCl pH 8.0, 100 mM NaCl, 5 mM EDTA, 0.5% SDS) containing protease inhibitors and incubated on ice for 10 min. Following centrifugation at 4 °C (12,000 rpm, 5 min), the supernatant was discarded and the pellet was retained. The pellet was resuspended in 1 mL of Triton dilution buffer, sonicated, and centrifuged. The resulting supernatant was precleared by incubation with 20 µL of Protein A/G beads (Thermo Fisher, USA) at 4 °C for 1 h with rotation to reduce nonspecific binding. A 100 µL aliquot was set aside as the Input control. The remaining chromatin sample was incubated overnight at 4 °C with rotation in the presence of 1–5 µg of STAT3 antibody (CST, USA) or control IgG (Santa Cruz, USA). Immune complexes were captured with Protein A/G beads and washed sequentially three times each with low-salt wash buffer (150 mM NaCl, 0.1% SDS, 1% Triton X-100, 2 mM EDTA, 20 mM Tris-HCl, pH 8.0), high-salt wash buffer (500 mM NaCl, 0.1% SDS, 1% Triton X-100, 2 mM EDTA, 20 mM Tris–HCl, pH 8.0), and LiCl wash buffer (100 mM Tris–HCl pH 7.5, 500 mM LiCl, 1% NP-40, 1% sodium deoxycholate). To reverse cross-linking, 200 µL of elution buffer and 5 µL of proteinase K (20 mg/mL) were added, followed by incubation at 65 °C for 6 h. DNA was purified using the QIAquick PCR Purification Kit (QIAGEN, Germany), dried, and eluted in 30 µL of EB buffer. For quantitative calibration, 10% of the Input DNA was processed in parallel.

For ChIP-qPCR assays, three biological replicates were performed for each experiment. Primer sequences used for ChIP-qPCR were as follows: TF-promoter-F, 5′-GTAAGACGACATCCCTGCCC-3′, TF-promoter-R, 5′-CTGGTAGAAACCGGCAACCA-3′ (located in −1 kb in the TF-promoter); β-actin-F, 5′-ATGGTGAGCTGCGAGAATAGC-3′, β-actin-R, 5′-GCCTTTTATGGTAATAACGCGG-3′.

### DIA mass spectrometry analysis

The nanoElute 2 liquid chromatography system (Bruker Daltonik, Bremen, Germany) was coupled to a time-of-flight high-resolution mass spectrometer (TOF HT) equipped with ion-mobility spectrometry and a quadrupole analyzer (Bruker Daltonik, Bremen, Germany). Proteins were harvested from HCCLM3 cells stably transfected with either TF overexpression (OE) or control vectors (OE-ctrl). After reconstitution in 0.1% formic acid, 200 ng of peptides were separated on an AUR3-15075C18 column (IonOpticks; 15 cm length, 75 μm internal diameter, 1.7 μm particle size, 120 Å pore size) using a 20-min gradient. The gradient started at 4% buffer B (80% acetonitrile with 0.1% formic acid), increased stepwise to 28% over 10 min, 44% in 3 min, and 90% in another 3 min, held at 90% for 1 min, and then re-equilibrated at 4% for 3 min. The flow rate was maintained at 300 nL/min with the column temperature set to 50 °C. DIA was performed in diaPASEF mode, with 22 precursor isolation windows of 40 Th width covering m/z 349-1229. To match the MS1 cycle time, a variable repetition scheme (2-5 steps) was applied within the 13-scan diaPASEF method. During PASEF MS/MS scanning, collision energy was linearly ramped based on ion mobility: from 59 eV at 1/K0 = 1.6 Vs/cm^2^ to 20 eV at 1/K0 = 0.6 Vs/cm^2^ (Mingyu Biotechnology, Shanghai, China).

### In vivo tumor growth and metastasis model

Stable-transfected HCCLM3 (overexpression) and Hep3B (knockdown) cell lines were implanted or injected to establish xenograft and lung/liver metastasis models in BALB/c nude mice (male, 4-6 weeks old). All mice were housed under pathogen-free conditions, randomly allocated to experimental groups. The study protocol was approved by the Medical Ethics Committee of Yangpu Hospital, School of Medicine, Tongji University, Shanghai, China (Approval No.: LL-2022-KXJS-001).

For the xenograft model, HCCLM3 (3 × 10⁶ per inoculation) or Hep3B cells (5 × 10⁶ per inoculation) from each group were subcutaneously injected into the left flank of mice (*n* = 6 per group). After four weeks, the mice were euthanized under anesthesia and tumors were excised. Tumor weight and volume were measured, and the specimens were fixed in phosphate-buffered neutral formalin for subsequent immunohistochemical analysis.

For the in vivo lung metastasis assay, HCC cells (1 × 10⁶) stably expressing different constructs were resuspended in 100 µL of serum-free DMEM and injected intravenously via the tail vein into BALB/c nude mice (male, 4-6 weeks old; *n* = 6 per group). Following a 6-week observation period, the mice were euthanized under anesthesia. Lungs were then harvested, fixed in phosphate-buffered neutral formalin, and processed for standard histology and metastatic nodule quantification.

For the in vivo liver metastasis assay, 2 × 10⁶ HCC cells stably expressing different constructs were resuspended in 100 µL of serum-free DMEM and injected into each BALB/c nude mouse (male, 6 weeks old). Prior to the procedure, mice were anesthetized with isoflurane. After shaving and disinfecting the left abdominal area with 75% alcohol, an approximately 1 cm lateral abdominal incision was made through the skin and peritoneum to expose the spleen. Using a 31G insulin syringe, the cell suspension was slowly delivered into the lower pole of the spleen (*n* = 5 per group). The peritoneum and muscle layer were closed with absorbable sutures, and the skin incision was secured using non-absorbable sutures. Following surgery, mice were placed on a heating pad until full recovery from anesthesia. Body weight and general condition were monitored every 2-3 days. Four weeks post-injection, the mice were euthanized under anesthesia. Livers were then harvested, fixed in phosphate-buffered neutral formalin, and processed for standard histological evaluation and quantification of metastatic nodules.

### Statistical analysis

For all experimental data, a minimum of three independent replicates were conducted. Results are expressed as mean ± standard deviation (SD). Statistical analyses were performed using GraphPad Prism software version 8 (GraphPad Software, San Diego, CA, USA). For comparisons between two groups, Student’s t-test was applied, while one-way analysis of variance was used for evaluating differences among three or more groups. Survival curves were generated using the Kaplan-Meier method, and group comparisons were assessed with the log-rank test. Associations between TF expression and clinicopathological characteristics were examined by chi-square test. A *P*-value of < 0.05 was considered statistically significant.

## Supplementary Information

Supplementary Material 1. Figure 1. Validation of survival-associated signature in independent TCGA-LIHC cohorts. Overall and recurrence-free survival were analyzed in the independent public TCGA-LIHC cohort. The corresponding Kaplan-Meier survival curves are presented. Figure 2. Establishment and functional characterization of stable TF-modified cell lines. Cellular immunofluorescence analysis was performed to detect EGFP‑positive HCC cells at 72 h post‑infection with lentiviral vectors mediating TF overexpression or knockdown. Puromycin (1μg/mL) was used for selection. TF-OE (HCCLM3) MOI=20, TF-KD (Hep3B) MOI=10. Figure 3. Gene ontology (GO) and KEGG pathway analysis of differentially expressed proteins. (a) Top 20 significant GO terms (biological processes) and (b) KEGG pathway terms associated with the identified differentially expressed proteins. Figure 4. PAR1-mediated signaling drives the primary intracellular effects of TF in HCC cells.(a) Western blot analysis showing PAR1 and PAR2 expression following TF overexpression and knockdown. (b and c) Cell proliferation was assessed using EdU assays in HCC cells. (d and e) Migration ability was evaluated by wound-healing assays; wound closure was measured at 0 h and 48 h post-scratch. (f and g) Representative images and quantitative analysis of transwell assays demonstrating the invasive capacity of HCC cells. (h and i) Three-dimensional spheroid formation assay illustrating spheroidizing ability of HCC cells; scale bar = 100 μm. (j) Cellular immunofluorescence staining of β-catenin and p-STAT3 in HCC cells; scale bar = 20 μm. (k) Western blot detection of β-catenin, STAT3, and p-STAT3 protein levels in cytoplasmic and nuclear fractions of HCC cells. ^*^P<0.05, ^**^P<0.01, ^***^P<0.001. Figure 5. Detection of AKT signaling pathway proteins in treated HCC cells. The expression levels of proteins associated with the PI3K/AKT/mTOR signaling pathway were analyzed by Western blot in HCC cell lines with stable TF overexpression or knockdown. GAPDH served as the loading control. Figure 6. Combined β‑catenin and STAT3 inhibition more effectively reduces ECM‑degradation and metastasis markers in HCC. The cytoplasmic expression of metastasis-associated proteins (MMP2, MMP9, Vimentin, E-cadherin, N-cadherin, and Snail) in HCC cells was assessed by western blot analysis. Figure 7. Pathway-specific agonist assays and co-immunoprecipitation show no direct interaction or cross-activation between β-catenin and STAT3 in HCC Cells. (a-b) Western blot analysis shows no reciprocal activation between the β-catenin and STAT3 signaling pathways. (c) Co-immunoprecipitation assays reveal no endogenous protein-protein interaction between β-catenin and STAT3, or between β-catenin and p-STAT3.

## Data Availability

All data that support the findings of this study are available from the corresponding authors upon reasonable request.

## Abbreviations

HCC: Hepatocellular carcinoma
TF: Tissue factor
PARs: Protease-activated receptors
CTCs: Circulating tumor cells
CTM: Circulating tumor microemboli
MVI: Microvascular invasion
PVTT: Portal vein tumor thrombus
ECM: Extracellular matrix
TME: Tumor microenvironment
OS: Overall survival
DFS: Disease-free survival
OE: Overexpression
KD: Knockdown
IHC: Immunohistochemistry
IF: Immunofluorescence
H&E staining: Hematoxylin and eosin staining
DIA-MS: Data independent acquisition mass spectrometry
PCNA: Proliferating cell nuclear antigen
MMPs: Matrix metalloproteinases
HBV: Hepatitis B virus
AFP: α-Fetoprotein
TNM: Tumor lymph node metastasis
SPF: Specific pathogen free
