# Supplementary material for: Tissue factor promotes vascular invasion and metastasis in hepatocellular carcinoma via combined activation of β-catenin/STAT3 signaling
Source: Mol Biomed. 2026 May 23;7:75. doi: 10.1186/s43556-026-00471-y (PMC13197542; doi:10.1186/s43556-026-00471-y)
Supplement: Supplementary file 1 — Supplementary Material 1. Figure 1. Validation of survival-associated signature in independent TCGA-LIHC cohorts. Overall and recurrence-free survival were analyzed in the independent public TCGA-LIHC cohort. The corresponding Kaplan-Meier survival curves are presented. Figure 2. Establishment and functional characterization of stable TF-modified cell lines. Cellular immunofluorescence analysis was performed to detect EGFP‑positive HCC cells at 72 h post‑infection with lentiviral vectors mediating TF overexpression or knockdown. Puromycin (1μg/mL) was used for selection. TF-OE (HCCLM3) MOI=20, TF-KD (Hep3B) MOI=10. Figure 3. Gene ontology (GO) and KEGG pathway analysis of differentially expressed proteins. (a) Top 20 significant GO terms (biological processes) and (b) KEGG pathway terms associated with the identified differentially expressed proteins. Figure 4. PAR1-mediated signaling drives the primary intracellular effects of TF in HCC cells.(a) Western blot analysis showing PAR1 and PAR2 expression following TF overexpression and knockdown. (b and c) Cell proliferation was assessed using EdU assays in HCC cells. (d and e) Migration ability was evaluated by wound-healing assays; wound closure was measured at 0 h and 48 h post-scratch. (f and g) Representative images and quantitative analysis of transwell assays demonstrating the invasive capacity of HCC cells. (h and i) Three-dimensional spheroid formation assay illustrating spheroidizing ability of HCC cells; scale bar = 100 μm. (j) Cellular immunofluorescence staining of β-catenin and p-STAT3 in HCC cells; scale bar = 20 μm. (k) Western blot detection of β-catenin, STAT3, and p-STAT3 protein levels in cytoplasmic and nuclear fractions of HCC cells. *P<0.05, **P<0.01, ***P<0.001. Figure 5. Detection of AKT signaling pathway proteins in treated HCC cells. The expression levels of proteins associated with the PI3K/AKT/mTOR signaling pathway were analyzed by Western blot in HCC cell lines with stable TF overexpressi [file 43556_2026_471_MOESM1_ESM.docx]

**Tissue factor promotes vascular invasion and metastasis in hepatocellular carcinoma via combined activation of β-catenin/STAT3 signaling**

Wen-chao Wang^1#^, Wei-dan Ji^2#^, Jun-yong Ma^3#^, Yun Pan^1#^, Lei Chen^4^, Xue-jing Lin^4^, Ying Chen^4^, Min Tang^1^, Hai-long Liu^1^
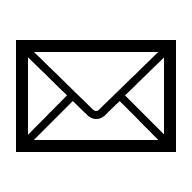
, Mou-bin Lin^1^
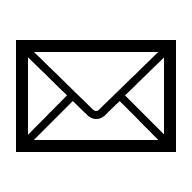
, Xiao-feng Zhang^3^
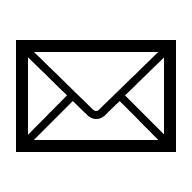
, Bin Sun^4,5^
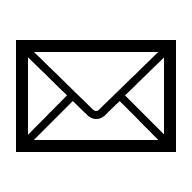


1. Department of General Surgery, Yangpu Hospital, Tongji University School of Medicine, Shanghai, 200090, P. R. China.

2. National Center for Liver Cancer, Navy Military Medical University, Shanghai, 200438, P. R. China.

3. Department of Hepatic Surgery, Eastern Hepatobiliary Surgery Hospital, Navy Military Medical University, Shanghai, 200438, P. R. China.

4. Center for Clinical Research and Translational Medicine, Yangpu Hospital, Tongji University School of Medicine, Shanghai, 200090, P. R. China.

5. Lead Contact

^#^These authors contributed equally to this work.


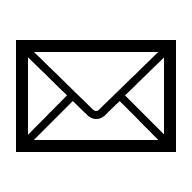
Corresponding authors:

Bin Sun, Center for Clinical Research and Translational Medicine, Yangpu Hospital, Tongji University School of Medicine, 450 Tengyue Road, Shanghai 200090, P. R. China. Email: [binsun@tongji.edu.cn](mailto:binsun@tongji.edu.cn).

Xiao-Feng Zhang, Department of Hepatic Surgery, Eastern Hepatobiliary Surgery Hospital, Navy Military Medical University, 225 Changhai Road, Shanghai 200438, P. R. China. Email: [zxf_ehbh@126.com](mailto:zxf_ehbh@126.com).

Mou-bin Lin, Department of General Surgery, Yangpu Hospital, Tongji University School of Medicine, 450 Tengyue Road, Shanghai 200090, P. R. China. Email: [1500142@tongji.edu.cn](mailto:1500142@tongji.edu.cn).

Hai-long Liu, Department of General Surgery, Yangpu Hospital, Tongji University School of Medicine, 450 Tengyue Road, Shanghai 200090, P. R. China. Email: [1500972@tongji.edu.cn](mailto:1500972@tongji.edu.cn).

**Supplementary Methods**

**Validation of prognostic significance using clinical study cohorts from public databases.**

Using the TCGAbiolinks package (version 2.32.0), we obtained FPKM transcriptomic data and corresponding clinical information for patients with liver hepatocellular carcinoma (LIHC) from the TCGA database. The optimal cutoff value for TF gene expression (in the TCGA database, the gene annotation for tissue factor (TF) is designated as F3) was determined with the maxstat algorithm implemented in the survminer package (version 0.4.9), based on which patients were stratified into high- and low-expression groups. Subsequently, Kaplan-Meier survival curves were generated using the survival package (version 3.7), and differences in survival outcomes between the two groups were evaluated via the log-rank test.

**Establishment of stable TF overexpressing and knockdown HCC cell sublines**

TF was stably overexpressed in the human HCC cell line HCCLM3 using a lentiviral construct (pcSLenti-EF1-EGFP-P2A-Puro-CMV-TF-3*Flag-WPRE), while knockdown was performed in Hep3B cells with a lentiviral shRNA vector (pSLenti-U6-shTF-CMV-EGFP-F2A-Puro-WPRE). All lentiviruses were designed and produced by OBIO Technology (Shanghai, China). The sequences for the overexpression construct (OE-TF) and three shRNAs targeting TF are provided in Supplementary Tables 2 and 3. Based on western blot analysis of TF knockdown efficiency, shRNA2 and shRNA3 (designated KD2 and KD3) were selected for further *in vitro* and *in vivo* studies. Corresponding controls included an empty overexpression vector (OE-ctrl) and a non‑targeting scramble shRNA (KD-ctrl). Each lentiviral vector carried EGFP and puromycin resistance genes. Infection efficiency was assessed via cellular immunofluorescence 72 h post‑infection.

**Western blotting assay**

Fresh paired HCC tissue specimens and HCC cells from various treatment groups were lysed using RIPA lysis buffer supplemented with 1% PMSF, 1% phosphatase inhibitor, and 1% protease inhibitor. Total cellular protein and nuclear protein were extracted following the protocol provided with a nuclear and cytoplasmic protein extraction kit (Beyotime, China), as previously outlined [1]. Details regarding the antibodies used are provided in Supplementary Table 4.

**Immunohistochemistry (IHC)**

IHC protocol for HCC tissue sections comprised sequential steps: tissue sectioning, antigen retrieval, blocking of non‑specific binding, incubation with primary and secondary antibodies, and chromogenic detection. The detailed procedures followed previously established methods [2], with antibody details provided in Supplementary Table 4.

Slides were digitized using an Aperio ScanScope FL slide scanner (Leica Biosystems, Canada). Positive DAB staining within the sample regions was identified and quantified using the HALO Multiplex IHC v3.0 algorithm (Indica Labs; Albuquerque, NM), which enabled statistical analysis of DAB-positive cells and evaluation of staining intensity per cell.

Further image analysis was performed with the HALO software suite (version 1.94.392, USA) through a custom algorithm developed by Freethinking Biotech (Jiangsu, China). Tissue slices were evaluated using histograms, paired scatter plots, and violin plots based on DAB H-scores. The expression of TF in HCC tissues was scored using the DAB H‑score, with the median value 65 used to classify samples into high‑TF (n=38) and low‑TF (n=25) groups.

**Cell proliferation assay**

EdU assay was performed to assess cell proliferation potential according to the manufacturer's protocol (Beyotime, China). Cells were seeded in 6-well plates at a density of 1×10⁵ per well and cultured for 24 h. Following this, 500 µL of 10 µM EdU working solution was added to each well and incubated for 2 h. After three washes with PBS, cells were fixed with 4% paraformaldehyde for 15 min, permeabilized with enhanced immunostaining permeabilization buffer for 15 min, and subsequently subjected to click reaction reagent for 30 min at room temperature in the dark. Finally, nuclei were counterstained with 1× Hoechst 33342. Fluorescence microscopy was used to visualize the results, and each experiment was performed in triplicate.

**Cell migration and invasion assay**

Wound-healing assays were performed to assess cellular migration ability. Briefly, cells were seeded in 6-well plates, and scratches were introduced upon confluence. Wound images were acquired at 0 h and 48 h post-scratch using a light microscope. The migration rate was quantified with Image‑J software.

Cell invasion was evaluated using Matrigel-coated transwell chambers (Corning, USA). Cells (1 × 10⁴) in 200 μL of serum-free medium were plated in the upper chamber, while the lower compartment contained 500 μL of complete medium with 10% FBS. After 48 h of incubation, non-invading cells on the upper membrane surface were gently removed. The membranes were fixed with 4% paraformaldehyde (Beyotime, China) and stained with 0.1% crystal violet (Beyotime, China). Invaded cells on the lower surface were counted under a microscope. All experiments were conducted in triplicate.

**Colony formation assay**

Cells were seeded at a density of 1 × 10³ per well in 6‑well plates and allowed to grow for two weeks. Following fixation with 4% paraformaldehyde, the colonies were stained with 1% crystal violet. Quantification of colony numbers was performed to evaluate proliferative capacity. All experiments were conducted in triplicate.

**CTC enrichment**

Density gradient centrifugation followed by magnetic sorting was employed to isolate circulating tumor cells (CTCs) from whole blood samples of hepatocellular carcinoma (HCC) patients. The specific protocols were conducted in accordance with our earlier published methodology [1].

**Three-dimensional (3D) culture *in vitro***

HCC cells were cultured in a three-dimensional (3D) model using Matrigel (BD Biosciences, USA), which provides an *in vivo*‑like growth environment. The procedure followed previously established methods [1]. Spheroid diameters were measured from bright‑field images acquired with an EVOS® FL Auto Imaging System (Thermo Fisher Scientific Inc, USA), with at least five independent fields analyzed per condition. Spheroids with a diameter ≥100 μm were considered normal. After two weeks of culture, the solidified Matrigel was dissolved with dispase (BD Biosciences, USA) at 37 ℃, gently washed with PBS, and the spheroids were collected for preparation of cell smears used in subsequent immunofluorescence assays.

**Cellular immunofluorescence (IF) detection**

The key steps for immunofluorescence (IF) staining of HCC spheroids comprised fixation, blocking of non-specific binding sites, incubation with primary antibodies, labeling with fluorescent secondary antibodies, and final visualization and image capture by fluorescence microscopy. Specific protocols followed previously established methods [1]. Details regarding antibodies used are provided in Supplementary Table 3.

**Treatment with β-catenin, STAT3 and PAR1 inhibitor**

The β-catenin inhibitor MSAB, the STAT3 inhibitor Stattic, and the PAR1 inhibitor Vorapaxar were obtained from MedChemExpress (MCE, USA). Following the manufacturer’s instructions, each compound was prepared as a 10 mM stock solution in DMSO. For *in vitro* cell-based assays, inhibitors were applied at a working concentration of 10 μM. In animal studies, inhibitors were administered intraperitoneally at 10 mg/kg, diluted in physiological saline, twice weekly for four weeks. Vehicle control groups received an equivalent volume of DMSO, corresponding to a final concentration of 0.1% (v/v) in culture medium (or in physiological saline for *in vivo* experiments).

**Treatment with β-catenin and STAT3 and PAR1 agonist**

SKL2001 (a β-catenin agonist) and ML115 (a STAT3 agonist) were obtained from MedChemExpress (MCE, USA). Following the manufacturer’s instructions, each compound was dissolved in DMSO to prepare 10 mM stock solutions. For all *in vitro* cellular assays, the agonists were applied at a working concentration of 10 μM. The vehicle control group was treated with an equivalent volume of DMSO, resulting in a final concentration of 0.1% (v/v) in the culture medium.

**Luciferase activity assay**

For cell culture under optimal growth conditions, seed cells in a 24-well plate one day prior to transfection. On the following day, perform transfection with the designated plasmids according to the experimental scheme. Following a 24-hour incubation, evaluate the expression of fluorescent marker genes (e.g., GFP) using fluorescence microscopy. Subsequently, apply the corresponding treatments to the cells and quantify luciferase activity with the Dual-Luciferase® Reporter Assay System (Promega, USA).

**Supplementary Figures**

**Supplementary Figure 1**


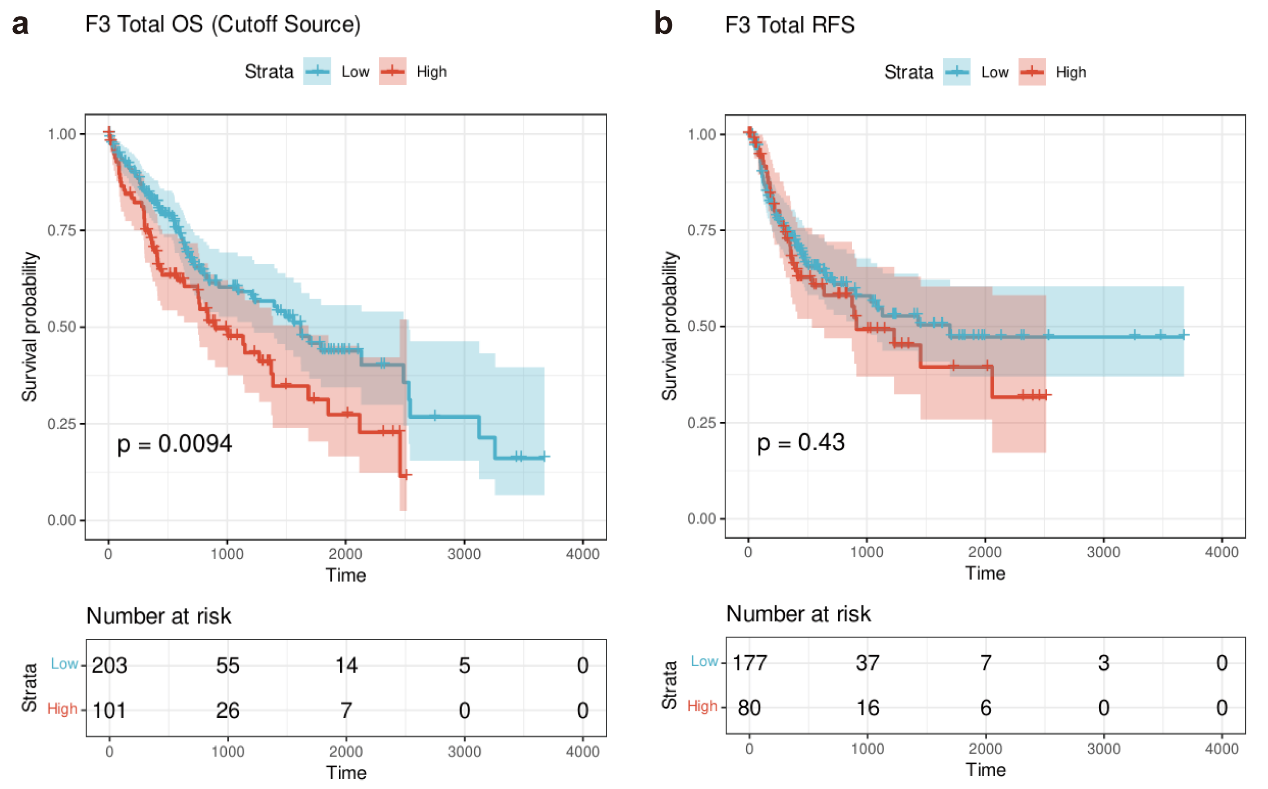


**Supplementary Figure 1. Validation of survival-associated signature in independent TCGA-LIHC cohorts.** Overall and recurrence-free survival were analyzed in the independent public TCGA-LIHC cohort. The corresponding Kaplan-Meier survival curves are presented.

**Supplementary Figure 2**


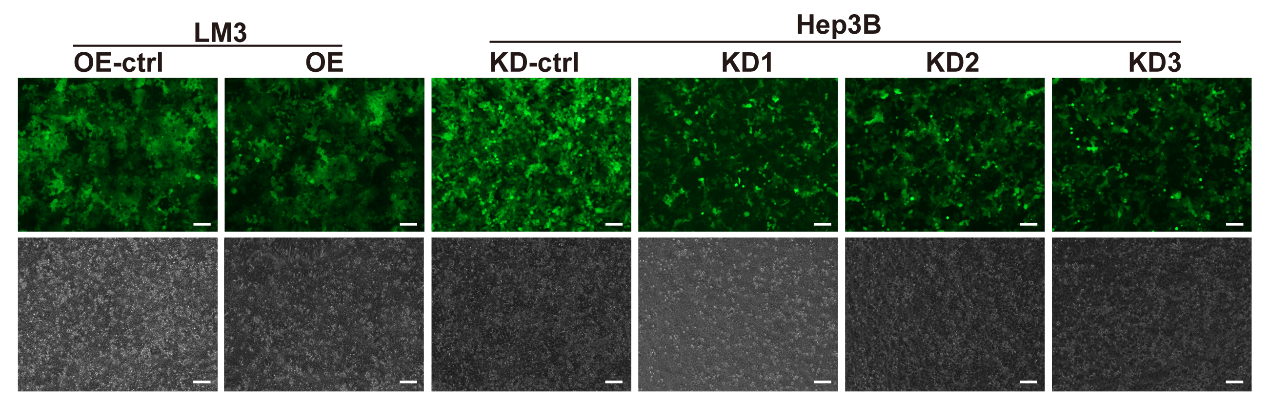


**Supplementary Figure 2. Establishment and functional characterization of stable TF-modified cell lines.** Cellular immunofluorescence analysis was performed to detect EGFP‑positive HCC cells at 72 h post‑infection with lentiviral vectors mediating TF overexpression or knockdown. Puromycin (1μg/mL) was used for selection. TF-OE (HCCLM3) MOI=20, TF-KD (Hep3B) MOI=10.

**Supplementary Figure 3**


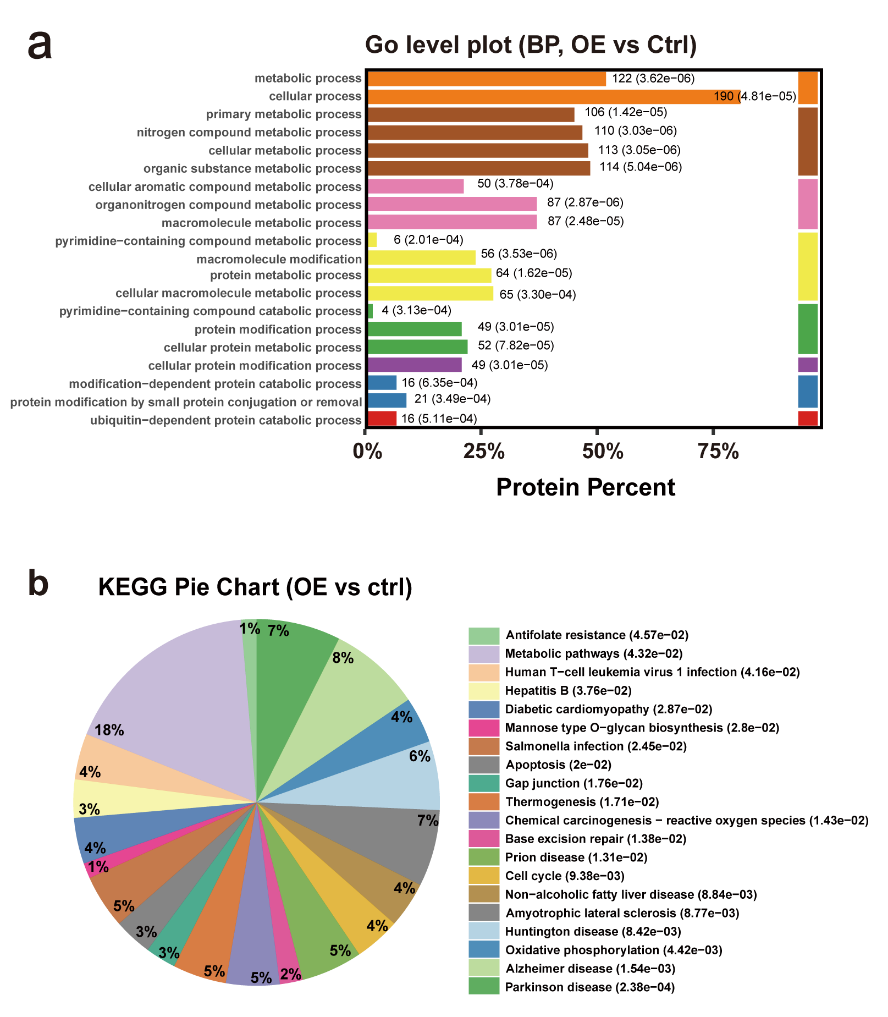


**Supplementary Figure 3.** **Gene ontology (GO) and KEGG pathway analysis of differentially expressed proteins.** (a) Top 20 significant GO terms (biological processes) and (b) KEGG pathway terms associated with the identified differentially expressed proteins.

**Supplementary Figure 4**


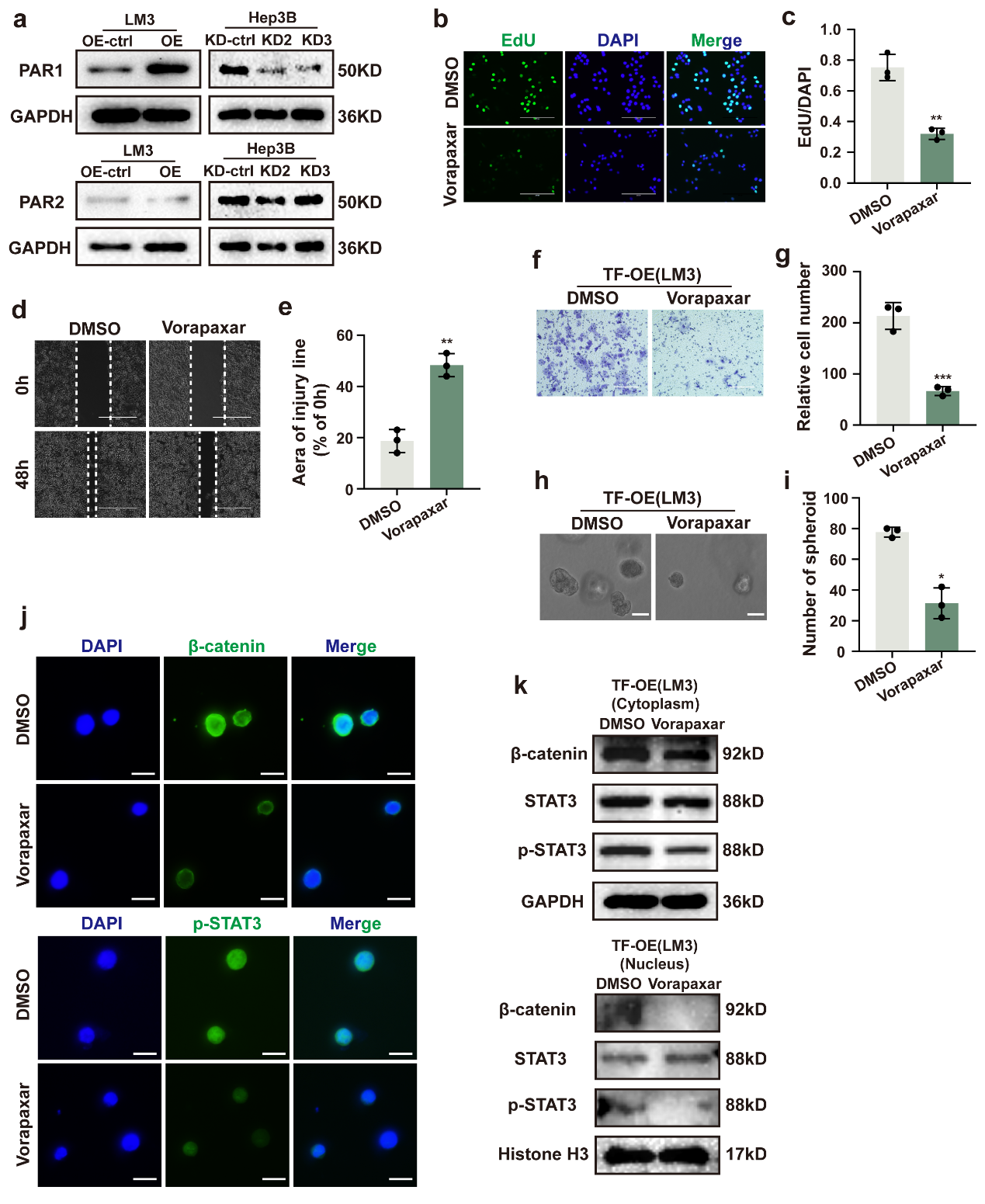


**Supplementary Figure 4. PAR1-mediated signaling drives the primary intracellular effects of TF in HCC cells.** (a) Western blot analysis showing PAR1 and PAR2 expression following TF overexpression and knockdown. (b and c) Cell proliferation was assessed using EdU assays in HCC cells. (d and e) Migration ability was evaluated by wound-healing assays; wound closure was measured at 0 h and 48 h post-scratch. (f and g) Representative images and quantitative analysis of transwell assays demonstrating the invasive capacity of HCC cells. (h and i) Three-dimensional spheroid formation assay illustrating spheroidizing ability of HCC cells; scale bar = 100 μm. (j) Cellular immunofluorescence staining of β-catenin and p-STAT3 in HCC cells; scale bar = 20 μm. (k) Western blot detection of β-catenin, STAT3, and p-STAT3 protein levels in cytoplasmic and nuclear fractions of HCC cells. ^*^*P*<0.05, ^**^*P*<0.01, ^***^*P*<0.001.

**Supplementary Figure 5**


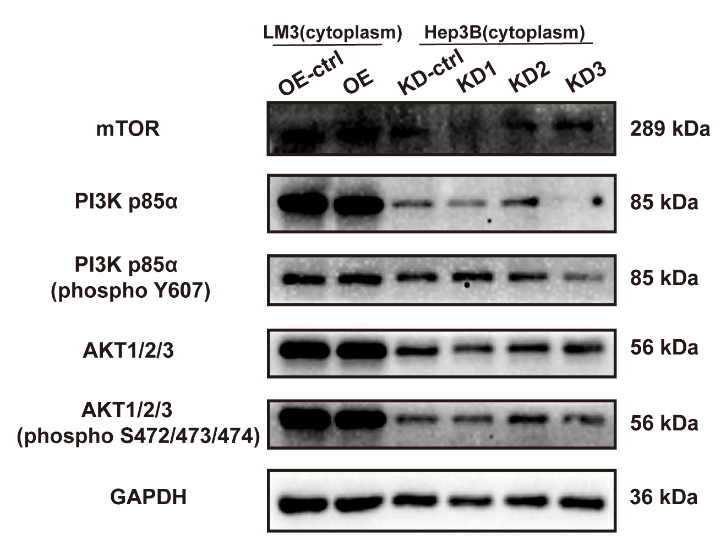


**Supplementary Figure 5. Detection of AKT signaling pathway proteins in treated HCC cells.** The expression levels of proteins associated with the PI3K/AKT/mTOR signaling pathway were analyzed by Western blot in HCC cell lines with stable TF overexpression or knockdown. GAPDH served as the loading control.

**Supplementary Figure 6**


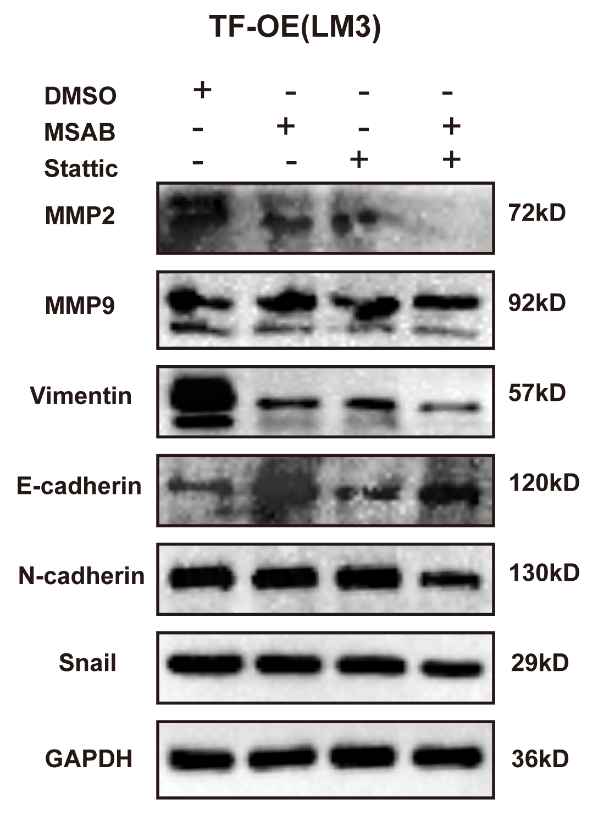


**Supplementary Figure 6. Combined β‑catenin and STAT3 inhibition more effectively reduces ECM‑degradation and metastasis markers in HCC.** The cytoplasmic expression of metastasis-associated proteins (MMP2, MMP9, Vimentin, E-cadherin, N-cadherin, and Snail) in HCC cells was assessed by western blot analysis.

**Supplementary Figure 7**


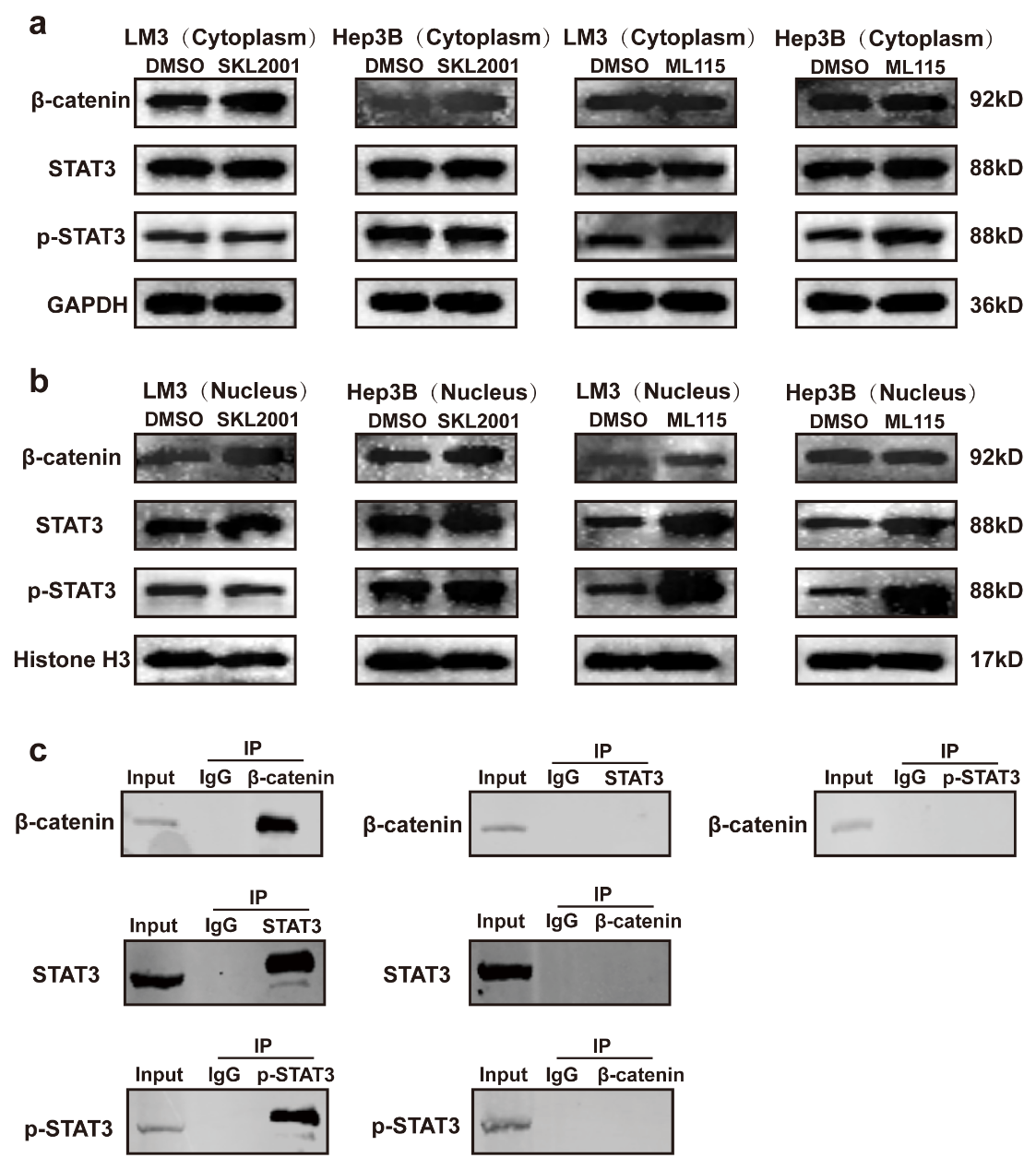


**Supplementary Figure 7. Pathway-specific agonist assays and co-immunoprecipitation show no direct interaction or cross-activation between β-catenin and STAT3 in HCC Cells.** (a-b) Western blot analysis shows no reciprocal activation between the β-catenin and STAT3 signaling pathways. (c) Co-immunoprecipitation assays reveal no endogenous protein-protein interaction between β-catenin and STAT3, or between β-catenin and p-STAT3.

**Supplementary Tables**

**Table S1. Associations between TF and the clinicopathological characteristics in 63 HCC cases.**

| **Variables** | **N**  **（N=63）** | | **TF**  **High** | **TF**  **Low** | **χ^2^** | ***p*** |
| --- | --- | --- | --- | --- | --- | --- |
| **Gender** |  | 38 | | 25 | 0.879 | 0.348 |
| Male | 58 | 34 | | 24 |  |  |
| Female | 5 | 4 | | 1 |  |  |
| **Age** |  |  | |  | 1.993 | 0.158 |
| ≤60 y | 55 | 35 | | 20 |  |  |
| >60 y | 8 | 3 | | 5 |  |  |
| **HBV status** |  |  | |  | 0. 659 | 0.417 |
| Yes | 51 | 32 | | 19 |  |  |
| No | 12 | 6 | | 6 |  |  |
| **AFP (ng/mL)** |  |  | |  | 0.466 | 0.495 |
| $\boldsymbol{>}$**20** | 53 | 31 | | 22 |  |  |
| $\boldsymbol{\leq}$**20** | 10 | 7 | | 3 |  |  |
| **Tumor size (cm)** |  |  | |  | 0.212 | 0.645 |
| $\boldsymbol{>}$**5** | 35 | 22 | | 13 |  |  |
| $\boldsymbol{\leq}$**5** | 28 | 16 | | 12 |  |  |
| **Tumor number** |  |  | |  | 3.618 | 0.055 |
| Multiple | 58 | 37 | | 21 |  |  |
| Single | 5 | 1 | | 4 |  |  |
| **Tumor encapsulation** |  |  | |  | 0.005 | 0.946 |
| Yes | 40 | 24 | | 16 |  |  |
| No | 23 | 14 | | 9 |  |  |
| **Vascular invasion** |  |  | |  | 5.999 | **0.014** |
| Yes | 42 | 27 | | 15 |  |  |
| No | 21 | 11 | | 10 |  |  |
| **Lymphatic metastasis** |  |  | |  | 4.473 | **0.029** |
| No | 43 | 22 | | 21 |  |  |
| Yes | 20 | 16 | | 4 |  |  |
| **TNM stage** |  |  | |  | 6.529 | **0. 011** |
| I-II | 41 | 20 | | 21 |  |  |
| III | 22 | 18 | | 4 |  |  |

**Abbreviation: TF, tissue factor; HCC, hepatocellular carcinoma; HBV, hepatitis B virus; AFP, α-fetoprotein; TNM, tumor lymph node metastasis. *P* < 0.05 was considered statistically significant.**

**Table S2. The target sequences of shRNA-TF**

| sh-h-TF-ctrl | CCTAAGGTTAAGTCGCCCTCG |
| --- | --- |
| sh-h-TF-1 | GTAGAAGATGAACGGACTTTA |
| sh-h-TF-2 | CACTGTTCAAATAAGCACTAA |
| sh-h-TF-3 | CCTGGCTATATCTCTACACAA |

**Table S3. The target sequences of overexpressed-TF**

ATGGAGACCCCTGCCTGGCCCCGGGTCCCGCGCCCCGAGACCGCCGTCGCTCGGACGCTCCTGCTCGGCTGGGTCTTCGCCCAGGTGGCCGGCGCTTCAGGCACTACAAATACTGTGGCAGCATATAATTTAACTTGGAAATCAACTAATTTCAAGACAATTTTGGAGTGGGAACCCAAACCCGTCAATCAAGTCTACACTGTTCAAATAAGCACTAAGTCAGGAGATTGGAAAAGCAAATGCTTTTACACAACAGACACAGAGTGTGACCTCACCGACGAGATTGTGAAGGATGTGAAGCAGACGTACTTGGCACGGGTCTTCTCCTACCCGGCAGGGAATGTGGAGAGCACCGGTTCTGCTGGGGAGCCTCTGTATGAGAACTCCCCAGAGTTCACACCTTACCTGGAGACAAACCTCGGACAGCCAACAATTCAGAGTTTTGAACAGGTGGGAACAAAAGTGAATGTGACCGTAGAAGATGAACGGACTTTAGTCAGAAGGAACAACACTTTCCTAAGCCTCCGGGATGTTTTTGGCAAGGACTTAATTTATACACTTTATTATTGGAAATCTTCAAGTTCAGGAAAGAAAACAGCCAAAACAAACACTAATGAGTTTTTGATTGATGTGGATAAAGGAGAAAACTACTGTTTCAGTGTTCAAGCAGTGATTCCCTCCCGAACAGTTAACCGGAAGAGTACAGACAGCCCGGTAGAGTGTATGGGCCAGGAGAAAGGGGAATTCAGAGAAATATTCTACATCATTGGAGCTGTGGTATTTGTGGTCATCATCCTTGTCATCATCCTGGCTATATCTCTACACAAGTGTAGAAAGGCAGGAGTGGGGCAGAGCTGGAAGGAGAACTCCCCACTGAATGTTTCA

**Table S4. The information of antibodies**

| **WB** | **Antibody** | **Company** | **Product number** | **Dilution** |
| --- | --- | --- | --- | --- |
|  | TF | abcam | 228968 | 1:1000 |
|  | PI3K/AKT panel | abcam | 283852 |  |
|  | (1) mTOR | abcam | STN-271336 | 1:10000 |
|  | (2) PI3K p85α | abcam | STN-400400 | 1:1000 |
|  | (3) PI3K p85α (phospho Y607) | abcam | STN-271387 | 1:1000 |
|  | (4) AKT1/2/3 | abcam | STN-271377 | 1:10000 |
|  | (5) AKT1/2/3 (phospho S472/473/474) | abcam | STN-271410 | 1:1000 |
|  | STAT3 | abcam | 68153 | 1:1000 |
|  | STAT3 (phospho Y705) | abcam | 76315 | 1:2000 |
|  | JAK2 | abcam | 108596 | 1:5000 |
|  | JAK2 (phospho Y1007+1008) | abcam | 32101 | 1:1000 |
|  | Wnt/β-catenin panel | abcam | 242226 |  |
|  | (1) Axin2 | abcam | STN-271258 | 1:1000 |
|  | (2) β-catenin | abcam | STN-274448 | 1:5000 |
|  | (3) GSK3β | abcam | STN-274441 | 1:5000 |
|  | (4) LEF1 | abcam | STN-271343 | 1:1000 |
|  | (5) CyclinD1 | abcam | STN-271333 | 1:10000 |
|  | Vimentin | abcam | 92547 | 1:1000 |
|  | MMP2 | abcam | 97779 | 1:1000 |
|  | MMP9 | abcam | 76003 | 1:1000 |
|  | E-cadherin | Proteintech | 60335-1-Ig | 1:2000 |
|  | N-cadherin | Proteintech | 66219-1-Ig | 1:5000 |
|  | Snail | Proteintech | 13099-1-AP | 1:1000 |
|  | Histone H3 | abcam | 1791 | 1:1000 |
|  | GAPDH | Proteintech | 60004 | 1:6000 |
| **IHC** | TF | abcam | 228968 | 1:500 |
|  | β-catenin | abcam | 32572 | 1:500 |
|  | Ki67 | Proteintech | 27309-1-AP | 1:2000 |
|  | PCNA | abcam | 92552 | 1:500 |
|  | STAT3 (phospho Y705) | abcam | 76315 | 1:100 |
|  | CD31 | abcam | 28364 | 1:50 |
| **IF** | TF | abcam | 228968 | 1:100 |
|  | panCK | abcam | 7753 | 1:50 |
|  | AF488-labeled Goat Anti-Mouse IgG (H+L) | Beyotime | A0428 | 1:200 |
|  | AF647-labeled Goat Anti-Rabbit IgG (H+L) | Beyotime | A0468 | 1:200 |
| **IP** | TF | abcam | 228968 | 1:30 |
|  | STAT3 | CST | 12640 | 1:100 |
|  | STAT3 (phospho Y705) | CST | 9145S | 1:100 |
|  | β-catenin | abcam | 32572 | 1:30 |

**References**

1. Sun B, Ji WD, Liu CY, Lin XJ, Chen L, Qian HH, et al. miR-2392 functions as tumour suppressor and inhibits malignant progression of hepatocellular carcinoma via directly targeting JAG2. Liver Int. 2022;42(7):1658-73. doi: 10.1111/liv.15284.

2. Li K, Zhang R, Wen F, Zhao Y, Meng F, Li Q, et al. Single-cell dissection of the multicellular ecosystem and molecular features underlying microvascular invasion in HCC. Hepatology. 2024;79(6):1293-309. doi: 10.1097/HEP.0000000000000673.
